# Genome-wide identification and characterization of parthenocarpic fruit set-related gene homologs in cucumber (*Cucumis sativus* L.)

**DOI:** 10.1038/s41598-023-29660-3

**Published:** 2023-02-10

**Authors:** Harleen Kaur, Pooja Manchanda, Pankaj Kumar, Rajinder Kumar Dhall, Parveen Chhuneja, Yiqun Weng

**Affiliations:** 1 School of Agricultural Biotechnology, College of Agriculture, Punjab Agricultural University, Ludhiana, 141004 India; 2 Department of Vegetable Science, College of Horticulture and Forestry, Punjab Agricultural University, Ludhiana, 141004 India; 3 USDA-ARS Vegetable Crops Research Unit, Department of Horticulture, University of Wisconsin, Madison, WI 53706 USA

**Keywords:** Computational biology and bioinformatics, Plant sciences

## Abstract

Cucumber (*Cucumis sativus* L.), a major horticultural crop, in the family Cucurbitaceae is grown and consumed globally. Parthenocarpy is an ideal trait for many fruit and vegetables which produces seedless fruit desired by consumers. The seedlessness occurs when fruit develops without fertilization which can be either natural or induced. So far, a limited number of genes regulating parthenocarpic fruit set have been reported in several fruit or vegetable crops, most of which are involved in hormone biosynthesis or signalling. Although parthenocarpic cucumber has been widely used in commercial production for a long time; its genetic basis is not well understood. In this study, we retrieved thirty five parthenocarpy fruit-set related genes (PRGs) from bibliomic data in various plants. Thirty-five PRG homologs were identified in the cucumber genome via homology-based search. An in silico analysis was performed on phylogenetic tree, exon–intron structure, cis-regulatory elements in the promoter region, and conserved domains of their deduced proteins, which provided insights into the genetic make-up of parthenocarpy-related genes in cucumber. Simple sequence repeat (SSR) sequences were mined in these PRGs, and 31 SSR markers were designed. SSR genotyping identified three SSRs in two polymorphic genes. Quantitative real-time PCR of selected genes was conducted in five cucumber lines with varying degrees of parthenocarpic fruit set capacities, which revealed possible association of their expression with parthenocarpy. The results revealed that homologs *CsWD40* and *CsPIN-4* could be considered potential genes for determination of parthenocarpy as these genes showed parental polymorphism and differential gene expression in case of parthenocarpic and non-parthenocarpic parents.

## Introduction

Cucumber (*Cucumis sativus* L.) belongs to the botanical family Cucurbitaceae that also includes several other economically important crops (cucurbits) such as melon (*Cucumis melo* L.), watermelon (*Citrullus lanatus* L.), squash/pumpkin (*Cucurbita* spp.), bitter gourd (*Momordica charantia* L.) and bottle gourd (*Lagenaria siceraria* L.)^1,2^. Being a predominantly monoecious crop, a successful fruit set in cucumber depends on conditions favourable to fertilization. The yield of cucumber is reduced by either absence of pollinators or under unsuitable environmental conditions, such as diffused light, high humidity and temperature^3^. Breeders have considered parthenocarpy as a trait to overcome the problem of poor fruit setting caused by unfavourable pollinating conditions^4^.

The production of parthenocarpic fruits is an attractive technique for the development of seedless fruits independent of pollination. Seedless fruits are favoured by breeders, cultivators as well as consumers. Moreover, parthenocarpic fruits are often firmer and fleshier than their seeded counterparts^5^. Therefore, development of parthenocarpy or production of fruits without seeds is desirable for cucumber breeding. Parthenocarpic fruits are formed when either the ovary develops directly without fertilization or when seed abortion occurs after ovary development without producing mature seeds^6^. Parthenocarpy is usually driven by genetic factors; however, it can be also induced by applying different phytohormones to young inflorescences^7^.

Parthenocarpy is a complex trait which is controlled by various phytohormones and multiple genes regulating the synthesis, transport and signalling of those phytohormones. It can also be induced artificially via exogenous application of plant growth hormones such as auxins (2, 4-dichlorophenoxyacetic acid; naphthaleneacetic acid), cytokinins (for example, forchlorfenuron, *N*-(2-Chloro-4-pyridyl)-*N*′-phenylurea) or CCPU, and; 6-benzylaminopurine), gibberellic acids (GAs) and brassinosteroids (BRs))^8^. Auxin was the first phytohormone to be recognized as an inducer of parthenocarpic fruit development in citrus and strawberry^4^. Auxin-related parthenocarpy could be affected by genes involved in auxin biosynthesis, transport, or signalling^9^. GA biosynthesis and signalling play an important role in parthenocarpic fruit set^10^. For example, overexpression and ectopic expression of the gene for the gibberellin 20-oxidase (an enzyme involved in the synthesis of bioactive gibberellic acid) leads to the production of parthenocarpic fruit in tomato (*Solanum lycopersicum)* and *Arabidopsis*^11^. Cytokinins have also been reported to promote the development of parthenocarpic fruit in a variety of species including watermelon (*Citrullus lanatus*), pear (*Pyrus pyrifolia*) and kiwi (*Actinidia deliciosa*)^12–14^. Ethylene (ET) affects parthenocarpic fruit set by working in partnership with auxin^3^. Abscisic acid (ABA) may act as an antagonist of gibberellic acid or auxin to attract and maintain the sleep state of the ovaries, possibly by suppressing their transition to fruit^15^.

Parthenocarpic expression can also be achieved via manipulation of genes involved in hormone signalling pathways. For example, transgenic tobacco and eggplants expressing the coding region of the *iaaM* gene from *Pseudomonas syringaepv. savastanoi*, under the control of the regulatory sequences of the ovule-specific *DefH9*(a MADS box) gene from *Antirrhinum majus*, showed parthenocarpic fruit development Expression of the *DefH9-iaaM* chimeric transgene occurs during flower development in both tobacco and eggplant^16^. Similarly, Yin et al.^17^ demonstrated that overexpression of the *DEFH9-IaaM* could stimulate parthenocarpy in cucumber. Ren et al.^9^ reported that the overexpression of *SlTIR1* resulted in parthenocarpy in tomato. Removing the function of negative regulators of auxin signalling encoded by *ARF8* (*AUXIN RESPONSE FACTORS*) and *ARF7* in *Arabidopsis* and tomato respectively also led to fertilization-independent fruit development^18,19^. Polycomb repressive complex 2 (PRC2) have been shown to contribute toward parthenocarpy^10^. *Arabidopsis* mutants defective in the PRC2-component genes have been linked to fertilization-independent seed development^20^. In *Arabidopsis,* PRC2 comprises of several genes. These genes consist of *MEDEA* (homolog of the *Drosophila melanogaster* gene *Enhancer of Zeste*), *FIS2* (homolog of the *Drosophila* gene *Suppressor of Zeste*), FIE (homolog of *Drosophila* extra sex combs), and *MSI1* (homolog of p55 in *Drosophila*)^20–22^.

Previous studies show a complex and confusing relationship between hormone responses during fruit set in cucumbers^8,23,24^. Recent studies on cucumber parthenocarpy have identified major loci (*parth2.1, parth5.1, parth7.1, parth6.1 and parth6.2*) and candidate genes (*CsARF19, CsWD40,* and *CsEIN1*)^24–26^. However, the key for assembling molecular players remains to be deciphered, and a global understanding of parthenocarpy processes is yet to be achieved. The present investigation aims to identify homologs of PRGs in cucumber with reference of PRGs which have already been reported in other crops such as *Arabidopsis*, tomato, fig and pear. The PRGs of various plants were retrieved from bibliomic data and used to search for PRG homologs in cucumber genome. The present investigation determination of chromosomal location, gene-structure prediction, identification of *cis-*regulating elements and conserved motifs, and physical and chemical analysis of the PRGs. Microsatellite markers/ simple sequence repeats (SSRs) associated with these PRGs were mined and validated in five cucumber genotypes. An expression study of the selected genes was performed through quantitative real-time PCR (qRT-PCR) in cucumber.

## Results

### Identification of cucumber PRGs

**Figure 1:**
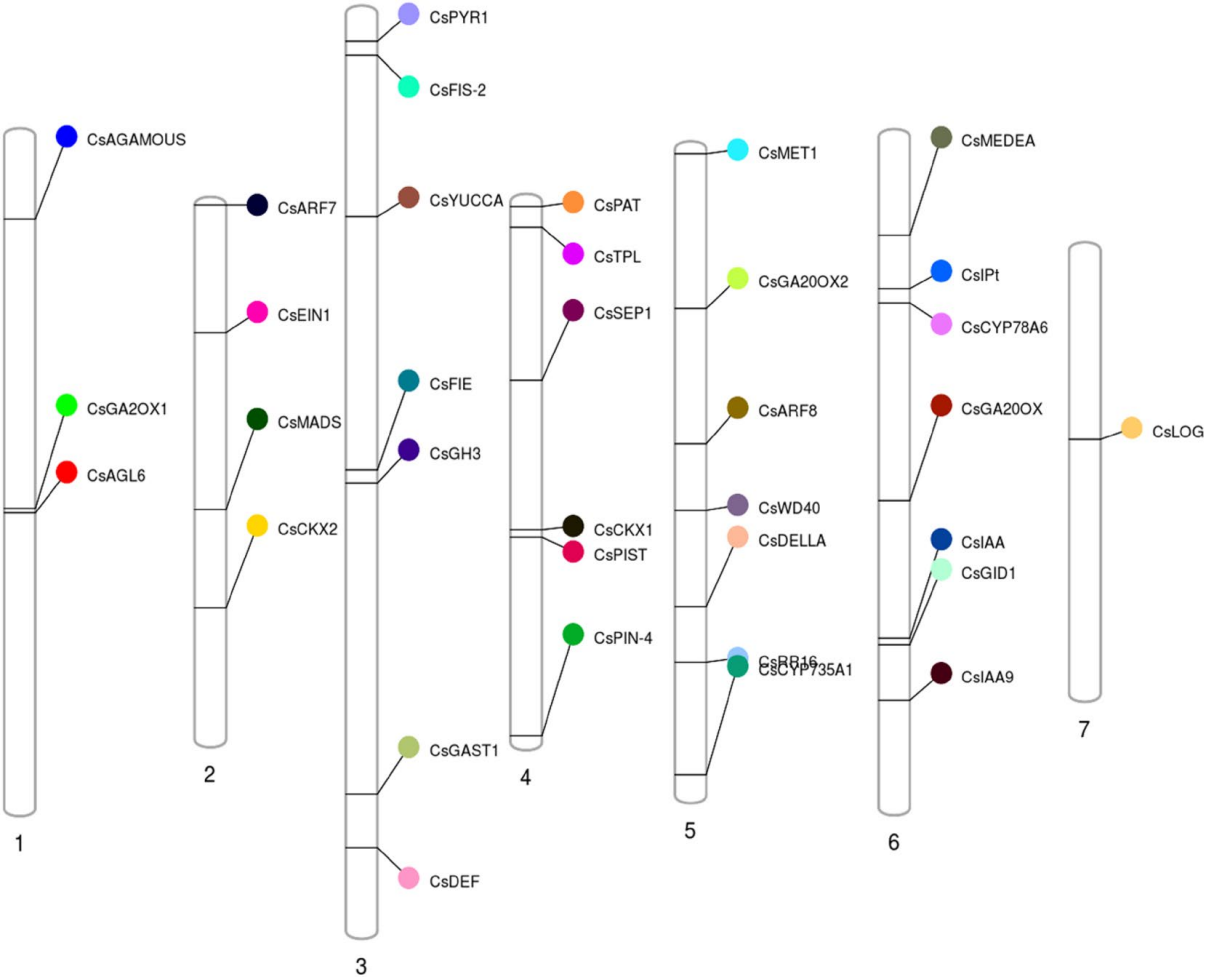
Graphical (scaled) representation of physical locations for parthenocarpy genes on cucumber chromosomes (numbered 1–7). Different colours of circles represent different genes.

Based on bibliomic data, 35 PRGs were identified from various crops including tomato (*Solanum lycopersicum*)*, Arabidopsis thaliana,* fig (*Ficus cracia*)*,* common pear (*Pyrus communis*)*,* grape (*Vitis vinifera*) and loquat (*Eriobotrya japonica*) (Table S1). The genes included *SlDELLA* (negative regulator of GA signalling)^27^, *SlARFs* (activation/inhibition of auxin responsive genes)^18,24^, *SlAGAMOUS/AGL* (MADS family transcription factor)^28,29^, *SlTPL* (Transducing family protein/WD40 repeat family protein)^30^, *SlPAT* (Synthesis of active gibberellic acid- natural parthenocarpy)^28,31^, *EjYUCCA* (for indole-3-pyruvate monooxygenase in auxin biosynthesis)^32^, *FcPYR* (ABA signalling pathway)^8,33^, *FcGID1* (*Gibberellin Insensitive Dwarf1—*gibberellic acid receptor)^34,35^, , *VvPISTILLATA/DEFICIENS* (MADS family transcription factors- controls petal and stamen floral organ identity)^10,36,37^, *CsLOG* (Lonely Guy enzyme- conversion of nucleotide precursors into active forms)^8,38^, *CsCKX* (Cytokinin oxidase- cytokinin degradation)^8,38^, *CsIPT* (Adenylateisopentenylatetransferase-cytokinin biosynthesis)^8,32^, *CsWD40* (WD-40 repeat family protein- cytokinin responses)^24^, *CsCYP735A* (for Cytochrome P450 monooxygenase- cytokinin biosynthesis)^8,38^ *AtFIE* (Fertilization Independent Endosperm)^10,22^, *AtFIS* (Fertilization Independent Seed)^10,20^, *AtMEDEA* (Polycomb group protein- transcriptional repression)^21,39^, *AtMET1* (methyl transferase- methylation of symmetric CpG residues)^39^, and *PbGA2ox* (Gibberellic acid oxidase)^40^. The gDNA sequences of them were used as queries to identify homologous genes in the cucumber genome (9930v2.0, https://cucurbitgenomics.org/), which are listed in Table 1. Genome-wide in silico analysis revealed the 35 PRGs were distributed across all 7 cucumber chromosomes with highest number of genes on chr 3, 5, 6 (n = 7), followed by chr 4 (n = 6), chr 2 (n = 4), chr 1 (n = 3) and chr 7 (n = 1) (Fig. 1).

**Table 1:** Physical and chemical properties of PRG proteins.

Gene name	Gene ID (9930v2.0)	Chromosome Location (9930v2.0)	Length (aa)	Intron number	PI value	Molecular weight (Da)	Subcellular location	Predicted pfam domain	Instability	Instability index	Aliphatic index	GRAVY
*CsYUCCA*	*Csa_3G133910*	3: 8,819,133–8,820,893	430	2	9.07	47,748.3	Cytoplasmic	Flavin-binding monooxygenase-like	Stable	39.49	88.16	-0.12
*CsDELLA*	*Csa_5G569350*	5:19,843,628–19,846,207	586	None	5.21	65,048.58	Nuclear	GRAS domain family	Unstable	45.78	83.58	–0.281
*CsMEDEA*	*Csa_6G055400*	6:4,253,629–4,256,457	182	1	9.13	19,395.85	-	-	Unstable	75.46	65.49	–0.298
*CsPIN-4*	*Csa_4G664490*	4:23,157,158–23,159,696	421	9	8.2	45,312.68	-	Membrane transport protein	Stable	38.91	128.74	0.712
*CsFIS2*	*Csa_3G017280*	3:1,803,363–1,815,236	433	13	5.95	49,762.18	Nuclear	VEFS-Box of polycomb protein	Unstable	55.5	70.65	–0.553
*CsPISTILLATA*	*Csa_4G358770*	4:14,523,954–14,527,317	143	5	5.62	17,033.33	Nuclear	K-box region	Unstable	54.06	62.03	–1.003
*CsDEFICIENS*	*Csa_3G865440*	3:36,197,413–36,201,586	206	6	9.82	24,519.6	Nuclear	SRF-type transcription factor	Unstable	40.4	84.66	–0.638
*CsFIE*	*Csa_3G416130*	3:19,778,725–19,783,358	370	12	6.02	41,621.51	Nuclear	WD domain, G-beta repeat	Unstable	48.19	85.54	-0.081
*CsGA20OX*	*Csa_6G351370*	6:15,776,533–15,779,194	378	2	8.02	42,691.6	-	2OG-Fe(II) oxygenase superfamily	Stable	36.78	74.55	–0.37
*CsMET1*	*Csa_5G002610*	5:197,583–217,191	1550	10	5.7	174,829.9	Nuclear	Cytosine specific DNA methyltransferase replication foci domain	Unstable	46	76.5	–0.506
*CsSEP1*	*Csa_4G126990*	4:7,740,614–7,747,713	184	6	6.61	21,085.82	Nuclear	K-box region	Unstable	46.65	78.42	–0.721
*CsARF7*	*Csa_2G000030*	2:17,946–33,432	1097	12	6.07	121,793.2	Nuclear	Auxin response factor	Unstable	67.92	72.56	–0.598
*CsARF8*	*Csa_5G315370*	5:12,784,117–12,806,542	783	13	6.08	87,589.45	Nuclear	Auxin response factor	Unstable	68.24	75.19	–0.469
*CsLOG*	*Csa_7G232550*	7:8,212,110–8,218,395	218	6	6.39	23,958.58	Cytoplasmic	Possible lysine decarboxylase	Unstable	47.1	90.73	–0.203
*CsIPT*	*Csa_6G095310*	6:6,578,003–6,589,862	962	12	5.83	107,532.4	Nuclear	CG-1 domain	Unstable	45.52	77.58	–0.491
*CsEIN1*	*Csa_2G070880*	2:5,520,557–5,526,964	740	5	7.08	82,674.61	Endoplasmic reticulum	Histidine kinase-, DNA gyrase B-, and HSP90-like ATPase	Unstable	40.9	109.16	0.159
*CsWD40*	*Csa_5G431540*	5:15,690,261–15,706,356	683	17	9.07	75,589.12	Nuclear	WD domain, G-beta repeat	Unstable	50.01	52.14	–0.73
*CsCYP735A1*	*Csa_5G644580*	5:27,133,440–27,138,283	419	4	9.24	47,515.44	Plasma membrane	Cytochrome P450	Unstable	49.17	96.11	–0.043
*CsRR16*	*Csa_5G603910*	5:22,259,935–22,261,511	233	3	5.38	25,556.67	Nuclear	Response regulator receiver domain	Unstable	90.54	76.91	–0.54
*CsPYR1*	*Csa_3G011650*	3:1,178,788–1,180,118	224	None	5.19	24,987.8	Multilocated	Polyketide cyclase / dehydrase and lipid transport	Unstable	45.68	82.23	–0.477
*CsCKX1*	*Csa_4G343590*	4:14,231,376–14,234,059	542	4	6.23	61,280.96	Vacuole	Cytokinin dehydrogenase 1, FAD and cytokinin binding	Stable	35.15	93.54	–0.13
*CsCKX2*	*Csa_2G362450*	2:17,471,628–17,475,204	434	3	6.07	47,744.3	Vacuole	Cytokinin dehydrogenase 1, FAD and cytokinin binding	Stable	30.22	93.85	–0.139
*CsMADS*	*Csa_2G277060*	2:13,207,264–13,207,992	187	None	9.18	21,583.89	Nuclear	SRF-type transcription factor	Unstable	54.96	84.49	–0.0349
*CsGA20OX2*	*Csa_5G172270*	5:6,923,672–6,925,695	373	2	6.4	42,493.42	–	2OG-Fe(II) oxygenase superfamily	Stable	29.84	74.72	–0.362
*CsGA2OX1*	*Csa_1G439830*	1:16,164,095–16,165,737	336	2	6.52	37,908.79	-	OG-Fe(II) oxygenase superfamily	Stable	38.02	90.24	–0.19
*CsIAA*	*Csa_6G454350*	6:21,728,038–21,730,868	441	4	5.82	47,744.41	Endoplasmic reticulum	Peptidase family M20/M25/M40	Stable	39.6	88.66	–0.021
*CsIAA9*	*Csa_6G497220*	6:24,438,988–24,442,688	380	4	6.48	41,601.57	Nuclear	AUX/IAA family	Unstable	44.46	68.76	–0.6
*CsAGAMOUS*	*Csa_1G033300*	1:3,602,044–3,605,237	317	9	4.78	35,904.06	Nuclear	-	Unstable	56.48	69.53	–0.714
*CsAGL6*	*Csa_1G446900*	1:16,330,489–16,331,245	152	1	5.17	17,127.73	Nuclear	-	Stable	32.15	87.83	–0.63
*CsTPL*	*Csa_4G006320*	4:1,069,354–1,076,252	1085	22	6.81	119,050.98	Nuclear	WD domain, G-beta repeat	Stable	39.92	79.29	–0.295
*CsGID1*	*Csa_6G476630*	6:22,003,109–22,008,005	320	1	5.95	35,387.36	-	alpha/beta hydrolase fold	Unstable	51.18	87.75	–0.123
*CsGAST1*	*Csa_3G841990*	3:33,853,617–33,854,721	103	3	9.03	11,314.23	Extracellular (secreted)	Gibberellin regulated protein	Unstable	51.19	46.41	–0.419
*CsPAT*	*Csa_4G000870*	4:200,973–204,864	475	9	6.77	50,928.46	Chloroplast	Aminotransferase class I and II	Stable	39.33	96.36	0.069
*CsCYP78A6*	*Csa_6G108440*	6:7,194,982–7,196,704	535	1	8.95	60,105.37	Plasma membrane	Cytochrome P450	Unstable	41.02	97.48	–0.022
*CsGH3*	*Csa_3G431430*	3:20,356,195–20,359,845	602	2	6.32	67,998.96	Cytoplasmic	GH3 auxin-responsive promoter	Unstable	43.95	88.37	–0.212

### Intron–exon structure of PRGs

**Figure 2:**
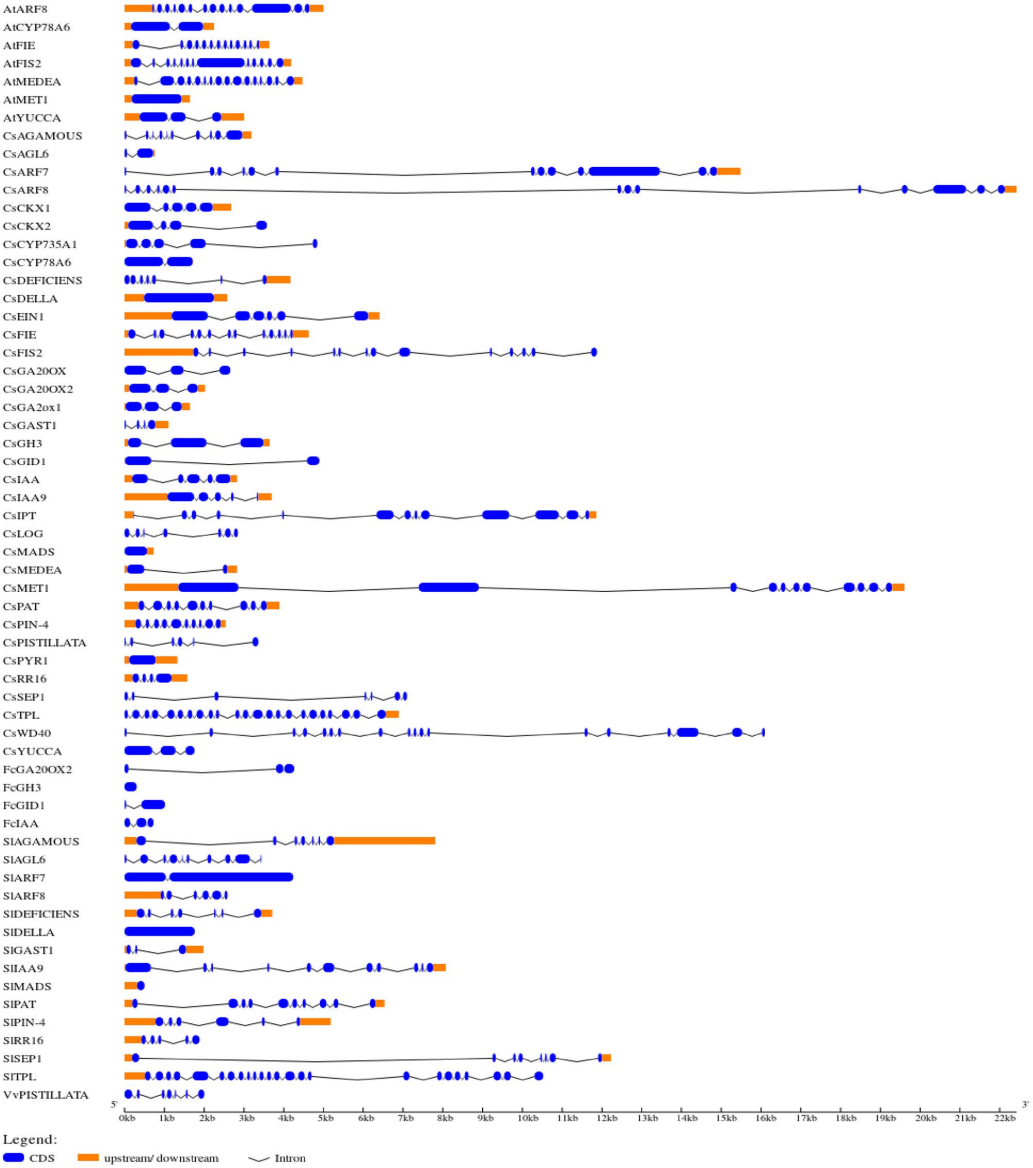
Intron–exon structures of cucumber PRGs and their reference genes from different crops. Exons and introns are shown by blue rectangles and thick black curved lines, respectively. Lengths of exons are fit to scale (At: *Arabidopsis thaliana;* Fc: *Ficus cracia;* Sl: *Solanum lycopersicum;* Vv: *Vitiv vinifera*).

Intron–exon structure of each PRG was predicted via the GSDS2.0 tool. The intron–exon organisation of each cucumber PRG and corresponding reference gene used as query is depicted in Fig. 2. In cucumber, the shortest genes included *CsMADS* and *CsAGL* (< 1 kb) while the longest one was *CsARF8* (> 22 kb). *CsTPL* had the most exons (22) while three genes (*CsDELLA, CsPYR1* and *CsMADS*) were intron-free with a single exon. The gene *CYP78A6* from *Arabidopsis* (*AtCYP78A6*) exhibited similar intron–exon organization as that of *CsCYP78A6*. The rest of the genes from *Arabidopsi*s (*AtARF8, AtFIE, AtFIS2* and *AtMEDEA*) showed great variation as compared with their cucumber homologs. The fig genes (*FcGA20OX2* and *FcGID1*) had the same number of exons as *CsGA20OX2* and *CsGID1* despite having dissimilar lengths. All the genes from tomato (except *SlDELLA*) showed significant variation in intron–exon structures as compared to cucumber homologs. The *PISTILLATA* gene had 6 intron in cucumber (*CsPISTILLATA*) while 7 introns in grape (*VvPISTILLATA*).

### Cis-regulatory element (CRE) analysis and identification of conserved motifs in PRGs

**Figure 3:**
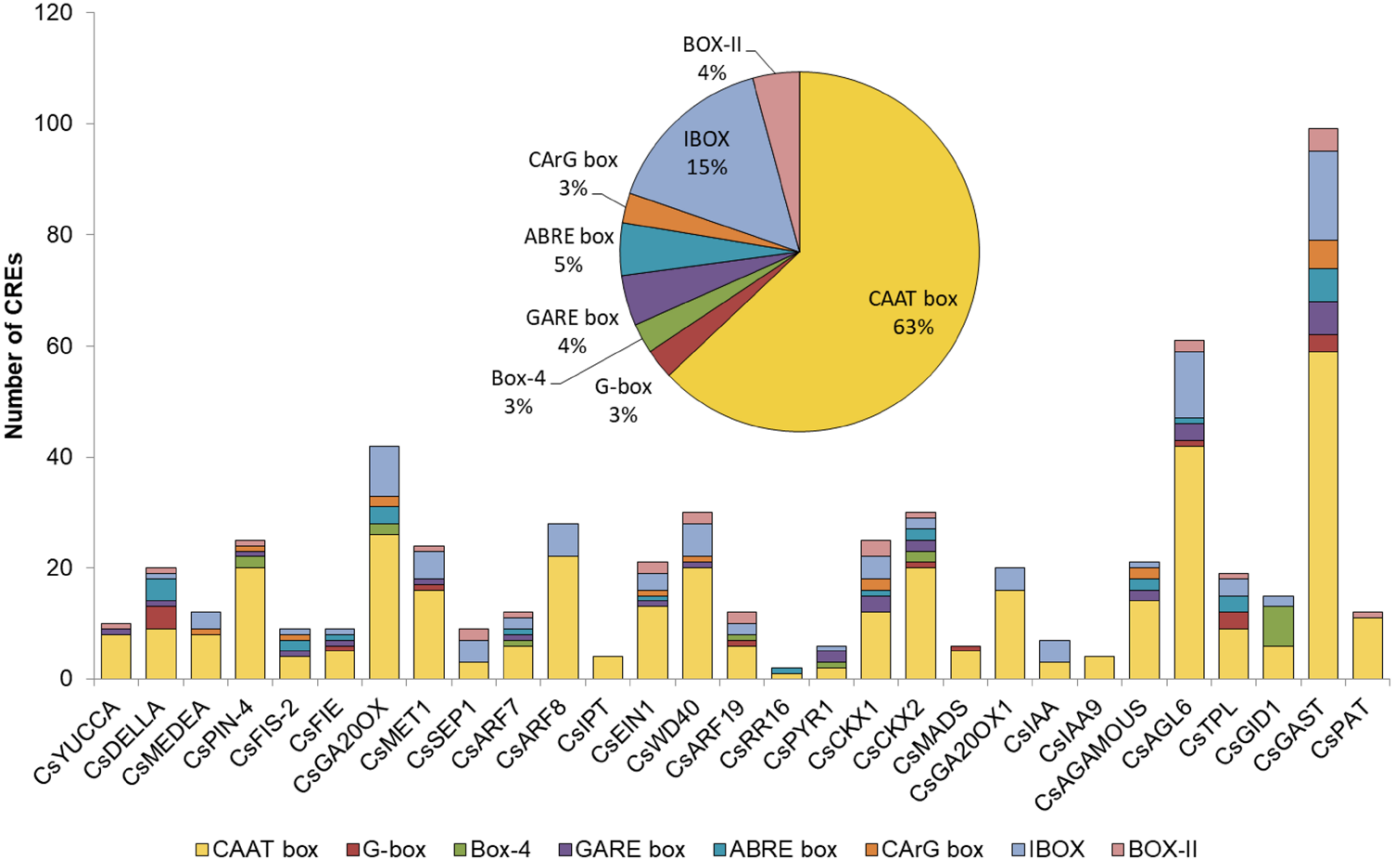
Distribution of various CREs on each gene (Insert: Abundance of each CRE).

The CREs responsible for parthenocarpy was examined in previous studies^41,42^. The promoter regions (> 300 bp) of the PRGs were analysed for CREs. Eight motifs were identified including the CAAT box, CArG box, G-box, Box-4, GARE box, ABRE box, Box-II and IBOX. The abundance and distribution of CREs in promoters of the PRGs are shown in Fig. 3. The CAAT box which is considered a core promoter element^43^ was the most abundant CRE present in all genes (63%). It helps in influencing the frequency of transcription initiation^44^. GARE and ABRE are gibberellin and ABA response elements respectively which had also been identified as TALE (three amino acid loop extension) gene members in pomegranate. G-box helps in regulating transcription of multiple genes^45^.

**Figure 4:**
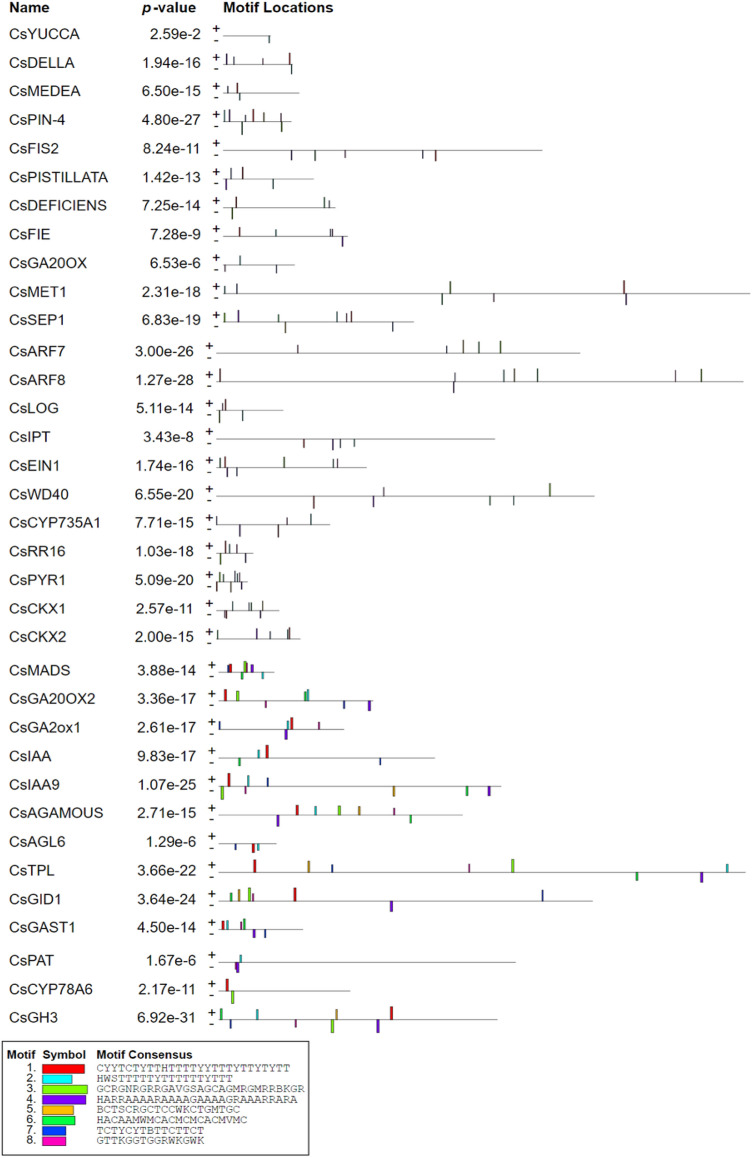
Conserved motifs in nucleotide sequences of PRGs in cucumber predicted using MEME suite. Different motifs are shown in different colours.

A motif sequence is a set of conserved amino acid residues which play an important role in protein functioning and are located within a certain distance from each other. These motifs help in elucidating the functions of uncharacterised proteins^46^. The conserved motifs were analysed via the MEME suite. In total, 8 motifs were predicted, as represented in Fig. 4 by solid blocks. The sequences of the motifs are given in Table 2. The most frequently occurring motif was motif 1 (CYYTCTYTTHTTTTYYTTTYTTYTYTT) and the least occurring motif was motif 5 (BCTSCRGCTCCWKCTGMTGC). The genes *CsPIN-4, CsSEP1, CsARF8* and *CsIAA9* had all eight motifs and the gene *CsYUCCA* had only one motif (motif 2). The gene *CsARF7* and *CsCKX2* had motifs only on the positive sense strand while the genes *CsYUCCA, CsFIS2, CsIPT* and *CsAGL6* had motifs only on the negative sense strand. The functional analysis of the motifs identified was performed using GoMo (Table 2). The GO (Gene ontology) terms were assigned to the motifs with high specificity (> 80%) except motifs 6 and 8. The motifs were categorized under molecular function and biological process under different GO terms. Based on motif annotation, motif 1 was annotated to be involved in polarity specification of adaxial/abaxial axis (GO:0,009,944), primary shoot apical meristem (GO:0,010,072) and ATP binding (GO:0,005,524). As parthenocarpy is closely regulated by plant hormones, the motifs were assigned GO terms in relation to general plant metabolism and phytohormone regulating pathways. The results indicated that these motifs may play roles in biological processes and metabolic functions such as ATP binding, transcriptional activity and cytokinin mediated signalling pathway (Table 2).

**Table 2:** List of eight motifs identified in cucumber PRGs.

Motif no	Motif	Specificity	GO term	*p-value*	GO term	GO name
1	CYYTCTYTTHTTTTYYTTTYTTYTYTT	100%	GO:0,005,524	2.652e-07	Molecular Function	ATP binding
			GO:0,010,072	1.750e-04	Biological Process	Primary shoot apical meristem specification
			GO:0,009,944	1.864e-04	Biological Process	Polarity specification of adaxial/abaxial axis
2	HWSTTTTTYTTTTTTYTTT	83%	GO:0,003,700	2.652e-07	Molecular Function	Transcription factor activity
3	GCRGNRGRRGAVGSAGCAGMRGMRRBKGR	100%	GO:0,005,524	2.652e-07	Molecular Function	ATP binding
4	HARRAAAARAAAAGAAAAGRAAARRARA	100%	GO:0,009,736	1.628e-04	Biological Process	Cytokinin mediated signalling pathway
5	BCTSCRGCTCCWKCTGMTGC	100%	GO:0,003,735	6.895e-06	Molecular Function	Structural constituent of ribosome
6	HACAAMWMCACMCMCACMVMC	No GO term found				
7	TCTYCYTBTTCTTCT	100%	GO:0,005,524	1.061e-04	Molecular Function	ATP binding
8	GTTKGGTGGRWKGWK	No GO term found				

### Phylogenetic analysis

**Figure 5:**
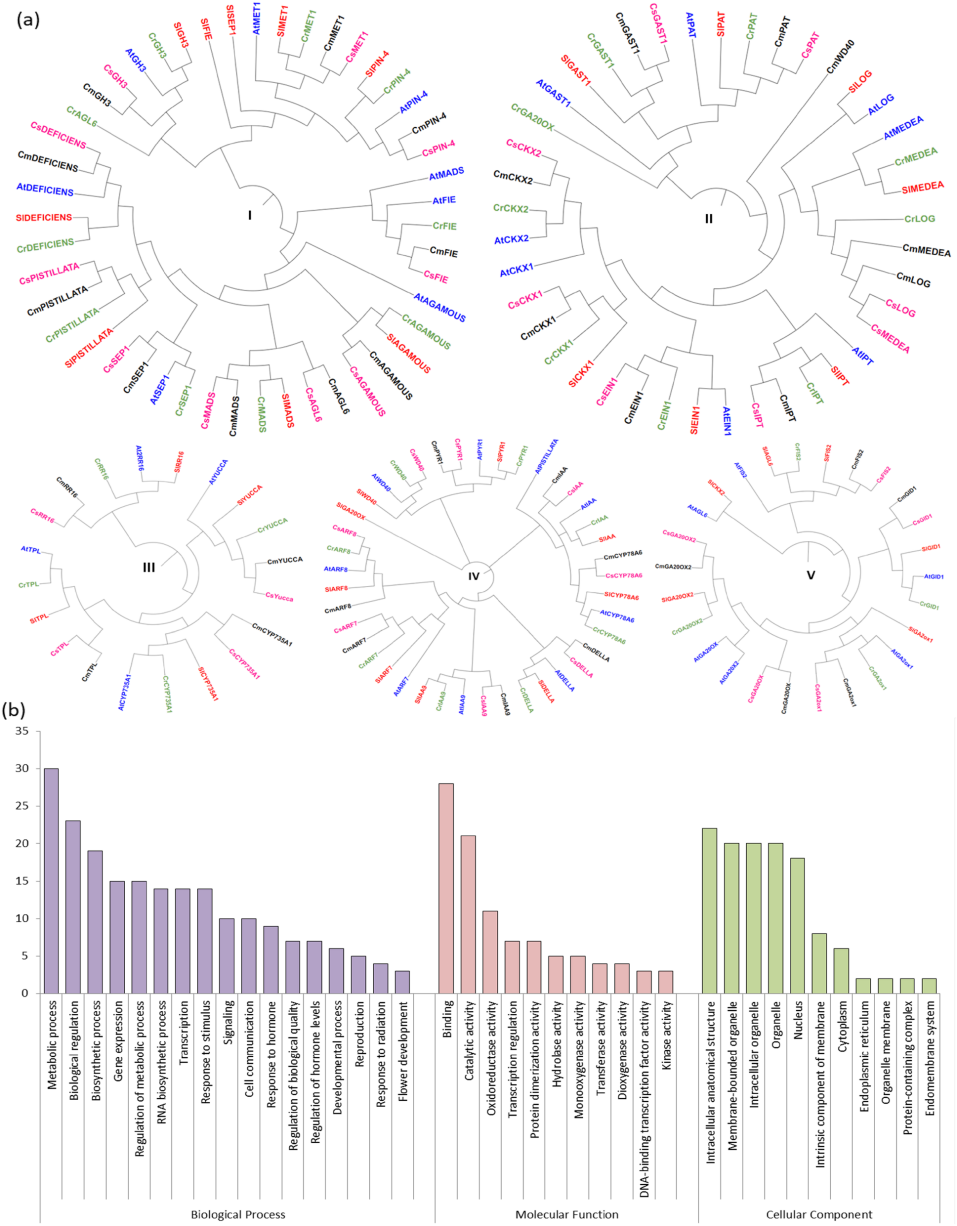
(**a**) Phylogenetic trees (homology groups I–V) showing relationship among 175 PRGs from *Arabidopsis* (At, blue), melon (Cm, black), citrus (Cr, green), cucumber (Cs, pink), and tomato (Sl, red) (**b**) Distribution of cucumber PRGs among three categories (cellular component, molecular functions and biological processes) via gene ontology analysis.

A phylogenetic tree was constructed using CDS sequences of 35 PRGs each from *Arabidopsis,* melon, cucumber, tomato and citrus (total 175 PRGs)*.* The phylogenetic tree was grouped in five homology groups on the basis of maximum likelihood in different species. In general, most of the cucumber genes were clustered with melon homologs and least related to tomato homologs. Based on the phylogeny, the genes were divided into five groups (I–V) with 47, 41, 20, 41 and 26 in each group respectively (Fig. 5a).

### Gene ontology

The protein sequences of the 35 PRGs were functionally annotated. The annotation was categorized into three categories based on three aspects: biological process, molecular function, and cellular component (Fig. 5b). The majority of the proteins belonged to the category biological process (GO:0,008,150) (Table S2). The proteins were involved in functions of metabolic process (GO:0,008,152) (n = 30), cellular process (GO:0,009,987) (n = 30), followed by cellular metabolic process (GO:0,044,237) (n = 29) and organic substance metabolic process (GO:0,071,704) (n = 26). The GO terms associated with molecular function (GO:0,003,674) predicted several categories including binding (GO:0,005,488) (n = 28) followed by catalytic activity (GO:0,003,824) (n = 21), organic cyclic compound binding (GO:0,097,159) (n = 22) and heterocyclic compound binding (GO:1,901,363) (n = 22). The cellular component category (GO:0,005,575) exhibited occurrence of proteins in various sub-cellular locations such as cellular anatomical entity (GO:0,110,165) (n = 25) followed by intracellular anatomical structure (GO:0,005,622) (n = 22). The functional enrichment of the genes was performed using Bonferroni method with threshold value of 0.05. The analysis categorized the genes into 4 categories as molecular function (n = 9), biological process (n = 40), cellular component (n = 1) and KEGG (Kyoto encyclopedia of genes and genomes; n = 2) (Fig S1). The detailed results of functional enrichment analysis have been provided in Table S3. Forty GO IDs related to biological process, nine GO IDs related to molecular function and one GO ID related to cellular component were identified. The highly enriched GO ID under biological process was biological regulation (GO:0,065,007) (n = 20) followed by regulation of macromolecule metabolic process (GO:0,060,255) and regulation of metabolic process (GO:0,019,222) with n = 14 each. In metabolic process, the maximum number of genes were assigned to double-stranded DNA binding (GO:0,003,690) with n = 7. Fourteen genes were placed under single GO ID of nucleus (GO:0,005,634) under cellular component. The KEGG pathway included plant hormone signal transduction and diterpenoid biosynthesis pathways.

### KEGG pathway

The KEGG pathway analysis revealed that the most genes were involved in plant hormone signal transduction and biosynthesis of secondary metabolites (Table 3). The genes encoding proteins cytokinin oxidase (CKX) and gibberellin oxidase (GAox) were involved in metabolic pathways and gene encoding indole acetic acid (IAA) and auxin response factors (ARF) were involved in plant hormone signal transduction. The gene *CsPAT* had a role in biosynthesis and metabolism of amino acids (tyrosine, phenylalanine) and alkaloid biosynthesis. The diterpenoid biosynthesis pathway involved the gibberellin oxidase genes and zeatin biosynthesis pathway involved the cytokinin oxidase and cytochrome-P450 monoxygenase genes. The detailed metabolic pathways are shown in Supplementary Fig. S2 (a-d). Among them, those involved in GA biosynthesis/signalling pathways included *DELLA, GA20OX, GA2OX, PAT* and *GID*. The metabolism of cytokinin is regulated by genes *IPT, CYP735A, LOG, RR* and *CKX*. The auxin pathways included genes such as *YUCCA* and *ARF*. The detailed functions of these genes were elucidated via functional enrichment and homology modelling.

**Table 3:** KEGG pathway analysis of genes from predicted to be involved in parthenocarpy.

Pathway	No of genes	Genes
Metabolic pathways	6	*CsMET1, CsGA20OX, CsGA20OX2, CsCYP735A1, CsYUCCA, CsPAT*
Biosynthesis of secondary metabolites	7	*CsCKX1, CsCKX2, CsGA2ox1, CsGA20OX, CsGA20OX2, CsCYP735A1, CsPAT*
Biosynthesis of amino acids	1	*CsPAT*
Cysteine and methionine metabolism	1	*CsMET1*
Tyrosine and phenylalanine metabolism	1	*CsPAT*
Tryptophan metabolism	1	*CsYUCCA*
Diterpenoid biosynthesis	3	*CsGA2ox1, CsGA20OX, CsGA20OX2*
Zeatin biosynthesis	4	*CsCKX1, CsCKX2, CsCYP735A1*
Alkaloid biosynthesis	1	*CsPAT*
mRNA surveillance pathway	1	*CsWD40*
MAPK signaling pathway	2	*CsPYR1, CsEIN1*
Plant hormone signal transduction	7	*CsIAA9, CsARF7, CsGH3, CsRR16, CsDELLA, CsPYR1, CsEIN1*

### Physical and chemical properties and homology modelling of PRG proteins

The physical and chemical properties of proteins encoded by the 35 PRGs were analysed including chromosomal location, length, PI (isoelectric point), molecular weight, instability, instability index, aliphatic index and GRAVY (Grand Average of Hydropathicity) index (Table 1). The length of proteins encoded by gene *CsMET1* was the highest while that of *CsGAST1* was the shortest. The proteins had an average PI value of 6.847 (ranged from 4.78 to 9.24). All the proteins had molecular weight higher than 2 kDa. The average molecular weight was 82,429.405 Da. The percent composition of essential amino acids in the proteins is given in Table S6. Some of the proteins appeared unstable in nature based on instability index of ProtParamExpasy > 40 except those encoded by *CsYUCCA, CsPIN-4, CsGA20OX, CsCKX1, CsCKX2, CsGA20OX2, CsGA2OX1, CsGA2OX2, CsIAA, CsAGL6, CsTPL* and *CsPAT*. The average aliphatic and GRAVY index were observed to be between 82.45 and − 0.3131, respectively.

**Figure 6:**
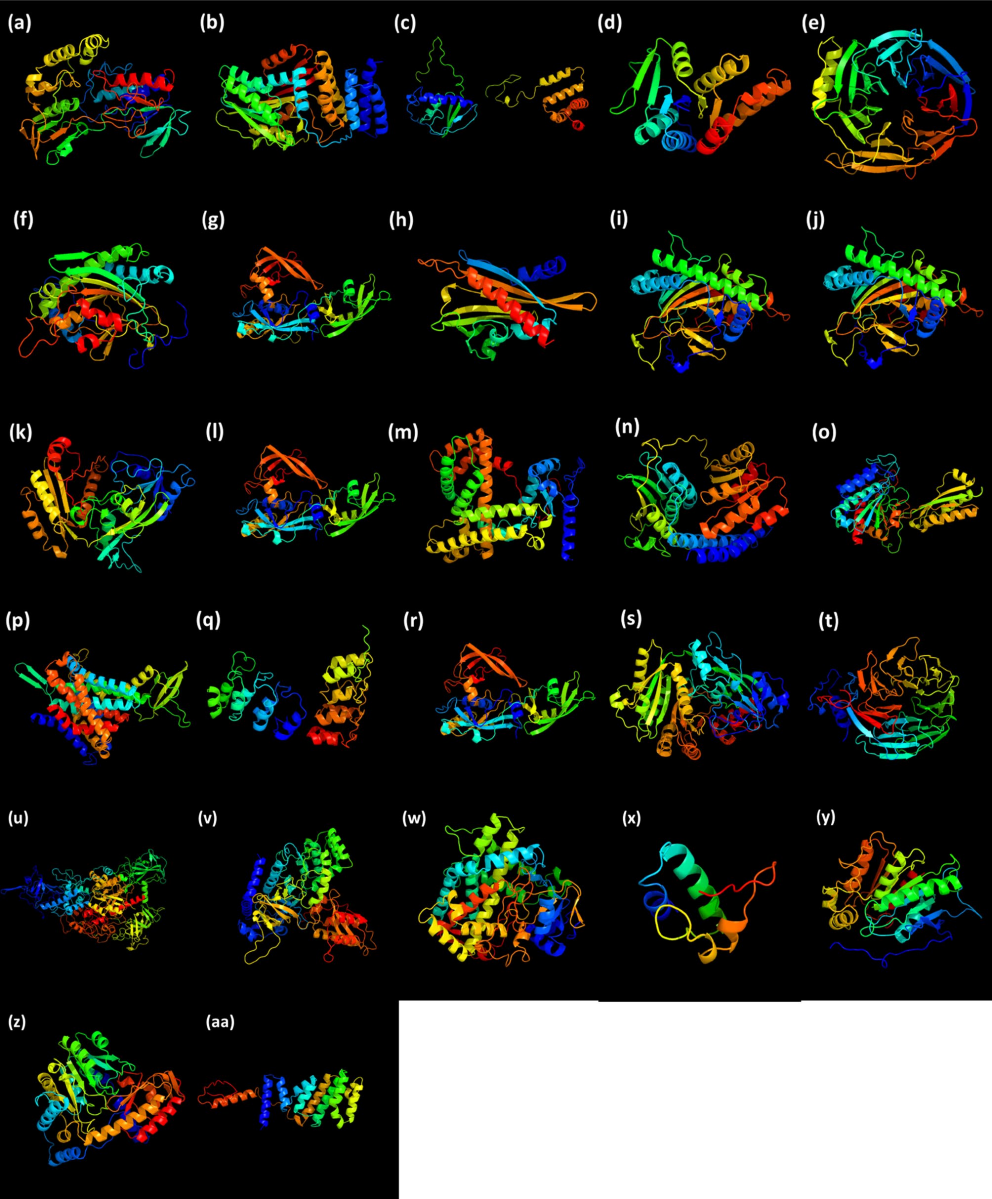
Predicted structures of proteins with 100% confidence level for the genes (**a**) *CsYUCCA* (**b**) *CsDELLA* (**c**) *CsFIS2* (**d**) *CsLOG* (**e**) *CsFIE* (**f**) *CsGA20OX* (**g**) *CsARF8* (**h**) *CsPYR1* (**i**) *CsGA2ox1* (**j**) *CsGA20OX2* (**k**) *CsCKX2* (**l**) *CsARF7* (**m**) *CsCYP735A1* (**n**) *CsEIN1* (**o**) *CsIAA* (**p**) *CsPIN-4* (**q**) *CsIPT* (**r**) *CsIAA* (**s**) *CsCKX1* (t) *CsWD40* (**u**) *CsMET1* (**v**) *CsGH3* (**w**) *CsCYP78A6* (**x**) *CsGAST1* (**y**) *CsGID1* (**z**) *CsPAT* (**aa**) *CsTPL*.

The structure of PRG proteins was predicted via homology modelling in Phyre2, which uses the alignment of Hidden Markov Models via HMM-HMM search to significantly improve the accuracy of alignment^47^. The template proteins used for modelling along with percentange of confidence for homology and conformational states are given in Table S4 and S5. The essential amino acid composition of the proteins has been provided in Table S6. Of the total proteins, structures of 27 proteins exhibited 100% confidence (Fig. 6). The prediction of the secondary structure of PRG by the protein homology revealed that the structures of the proteins predominantly comprised of α-helices (8.38–69.23%), extended strands (2.27–23.78%), β-turns (0–9.19%) and random coils (15.53–66.02%) (Table S5). The only protein without any β-turn was encoded by gene *CsSEP1.* The proteins encoded by genes *CsGA2ox1* and *CsGA20OX2*; and *CsARF7* and *CsARF8* shared similar structure (composition of α and β structures) but were different in their essential amino acid composition (Table S6). The functional role of the proteins was also determined during the homology modelling. The proteins were identified under several PDB header and PDB molecules (Table S4). Most of the proteins showed the functions in components of either plant development and signalling pathways such as sepallata, or auxin response factors or as constituents of enzymes involved in metabolism of plant hormones such as gibberellins, cytokinins and indole-acetic acid. The overall secondary structures of PGR proteins gave insights into the different domains such as catalytic domain, binding domain, N-terminal and C-terminal along with the presence of α and β structures. The homology modelling might help in the future to develop point mutation, and identifying master regulator for regulation. These PRG proteins could help to achieve specific targets by their use in genetic engineering tools such as CRISPR and RNAi (RNA interference) studies. Hence, all the predicted protein structures could be considered highly reliable offering a preliminary basis for understanding the molecular function of parthenocarpy-related proteins along with regulation by other factors.

### Protein–protein interaction (PPI) network of PRG homologs

**Figure 7:**
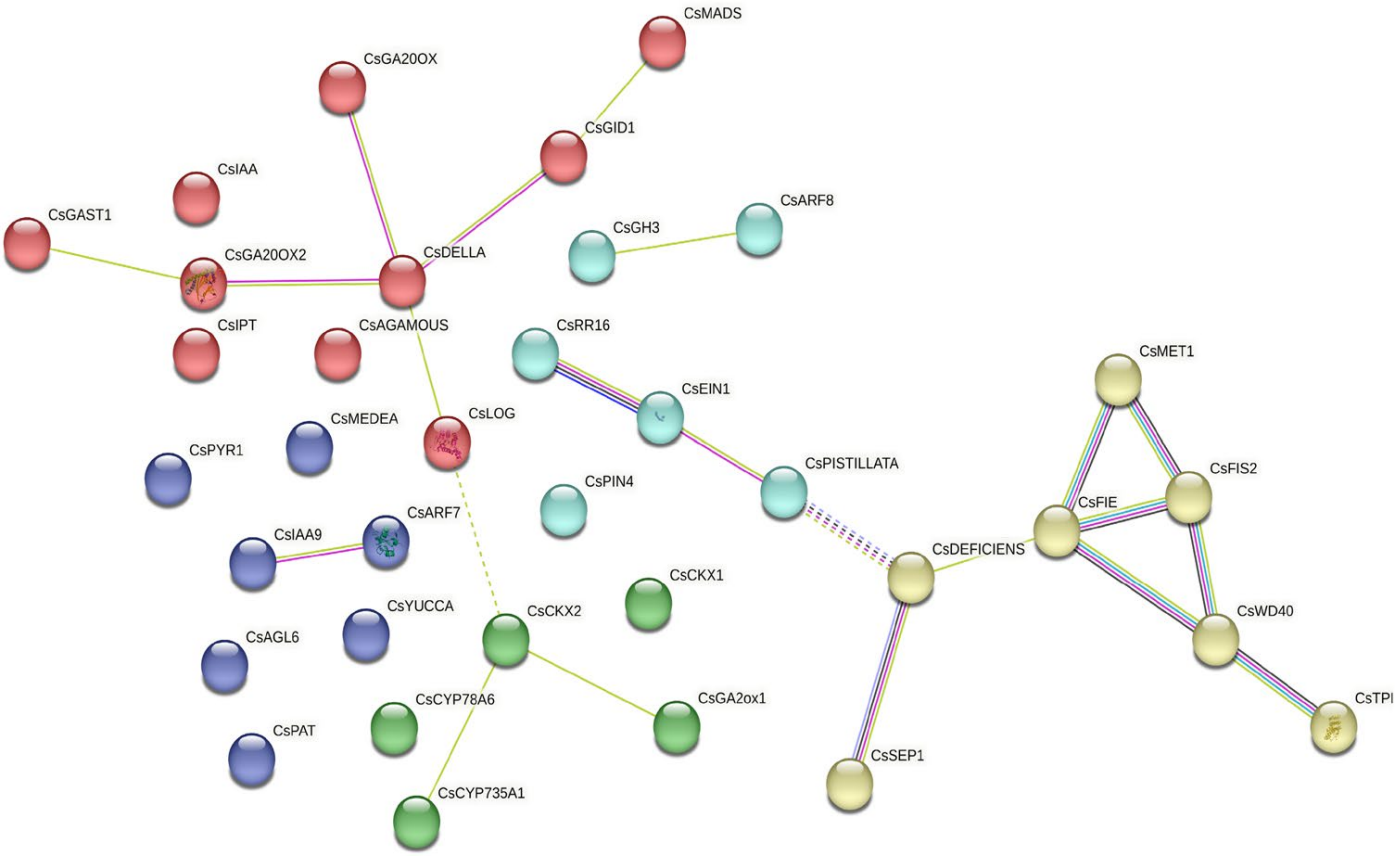
Protein–protein interactions (PPI) of parthenocarpy related genes constructed using Cytoscape. Dotted lines represent edges between clusters.

The PPI network for the PRGs was retrieved through STRING and clustered via k-means clustering. Three interconnected networks were identified (Fig. 7). The network constituted 35 nodes and 22 edges with average node degree of 1.26 and interaction score > 0.4. Most of the interactions generated were based on either text mining or experimentally derived depicted by green and pink lines respectively. The other node colours represented various interactions which included teal (from curated databases), blue (gene co-occurrence), dark green (co-expression) and lilac (protein homology). The clustering divided the proteins into five clusters with average local clustering coefficient of 0.41. The proteins in the same cluster shared similar biological function, such as red (response to gibberellin), yellow (WD repeat-containing domain superfamily), green (cytokinin metabolism), cyan (phosphoproteins) and blue (proteins without any significant clustering co-efficient). The results indicated that the proteins encoded by genes *CsDEFICIENS* and *CsDELLA* occupied the central positions in two different clusters. The *CsDEFICIENS* interacted with *CsPISTILLATA, CsSEP1* and *CsFIE*. Both PISTILLATA and SEP1 (SEPALLATA) are MADS box transcription factor proteins^48,49^. The protein encoded by *CsDELLA* was related to proteins having functions as gibberellin oxidase (encoded by *CsGA20OX, CsGA2OX, CsGA20OX2*), cytokinin oxidase (encoded by *CsCKX2, CsCYP735A2*), Gibberellin Insensitive Dwarf receptor (encoded by *CsGID*) and lonel guy enzyme (encoded by *CsLOG*). The *CsAux/IAA* and *CsARF8* were connected with *CsIAA9* and *CsGH3* respectively.

### Evaluation of SSR markers

**Figure 8:**
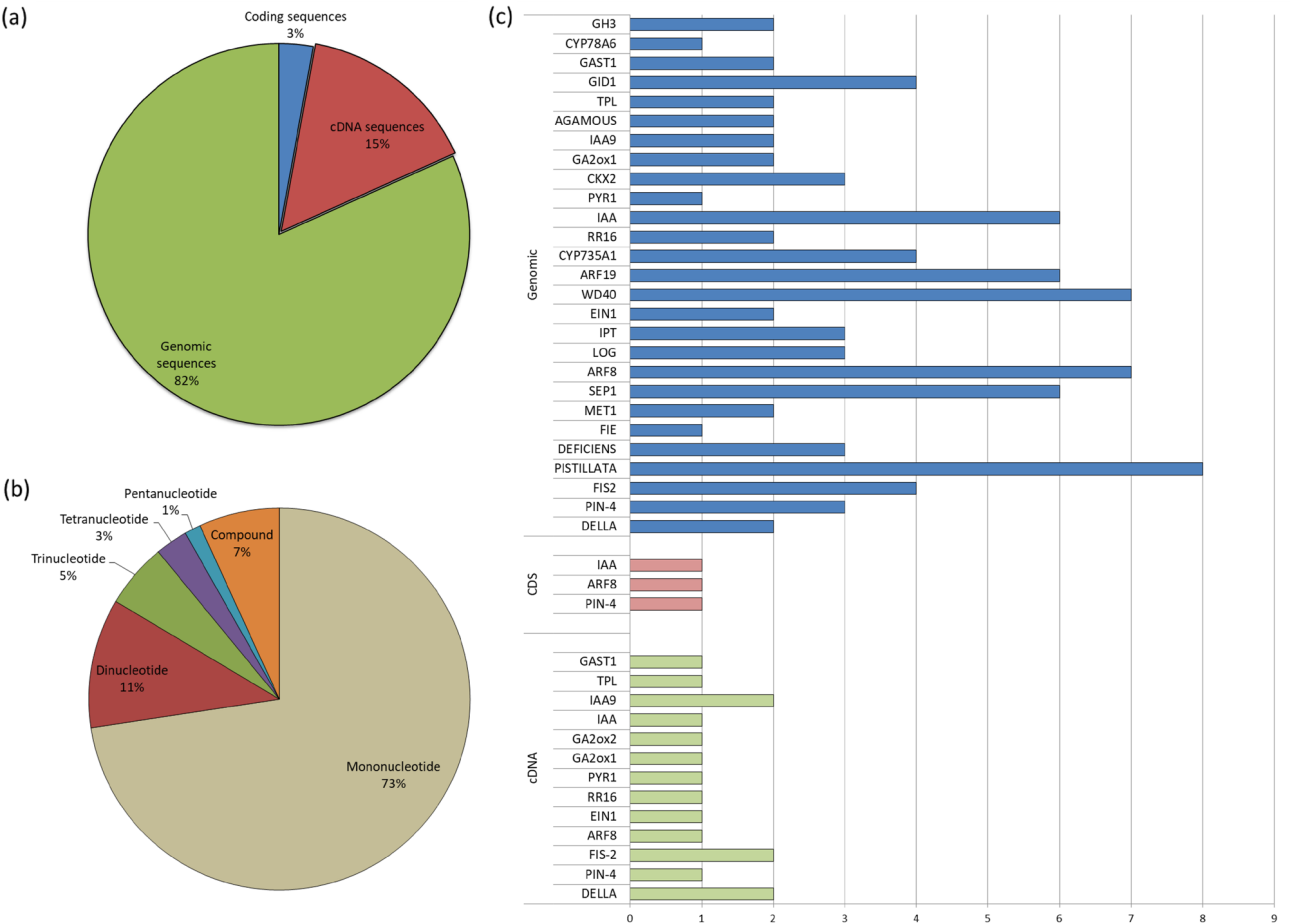
**(a)** Distribution of SSR markers among genomic, cDNA and coding sequences of PRGs **(b)** Abundance of mono-, di-, tri-, tetra-, penta-nucleotide repeats and compound SSRs in genomic sequences of PRGs. **(c**) Number of SSRs discovered from genomic, cDNA and coding sequences of various PRGs.

**Figure 9:**
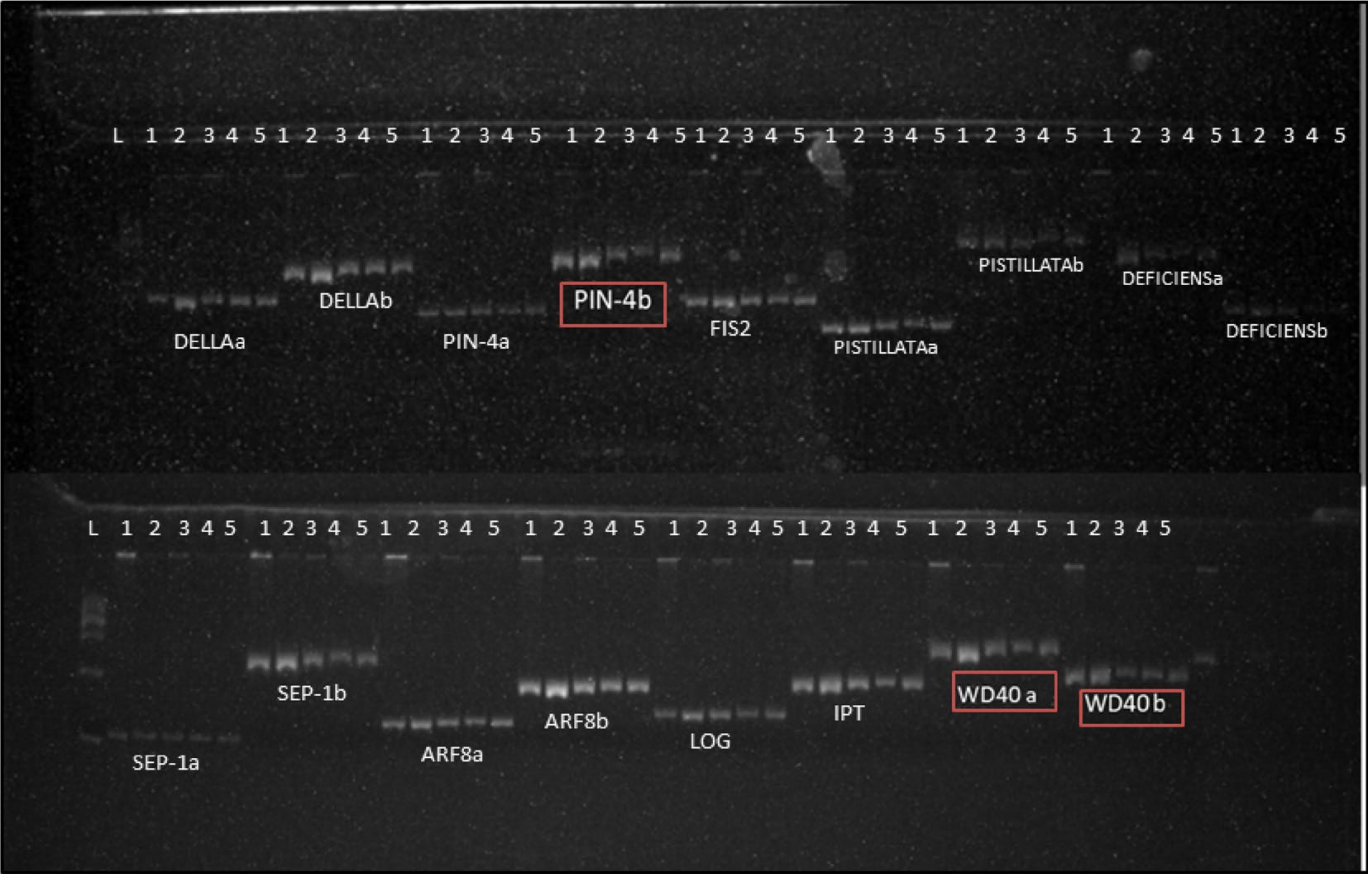
PAGE image showing parental polymorphism (L: 100 bp ladder; 1: Punjab Naveen; 2: Gy-14; 3: AVCU1303; 4: Punjab Kheera-1; and 5: PBRK5). Polymorphic markers are shown in red box.

Simple sequence repeats (SSRs) were mined in the genomic, coding and cDNA sequences of PRGs. One hundred and four SSRs were identified, which are presented in Table S7. The distribution of the 104 SSRs across genomic, cDNA and coding sequences are shown in Fig. 8a. Most SSRs are harboured in genomic sequences which included mono-, di-, tri-, tetra- and penta-nucleotide repeats and in compound formation (Fig. 8b). The most abundant form of SSRs was as mononucleotide repeats followed by dinucleotide repeats and compound SSRs. The distribution of SSRs across individual PRG in case of genomic, cDNA and coding sequences is shown in Fig. 8c. The maximum number of SSRs were detected in *PISTILLATA* followed by *WD40* and *ARF8*. The SSR markers were validated via PCR amplification and product separation via PAGE (Polyacrylamide gel electrophoresis). A total of 31 pairs of primers were designed based on these markers (Table S8). Out of the total, three primers for the genes *CsPIN-4* and *CsWD40* showed polymorphism within the parental genotypes i.e. Punjab Kheera-1, Gy-14, PBRK5, Punjab Naveen and AVCU1303 (Fig. 9).

### Expression analysis of PRGs in five cucumber lines

**Figure 10:**
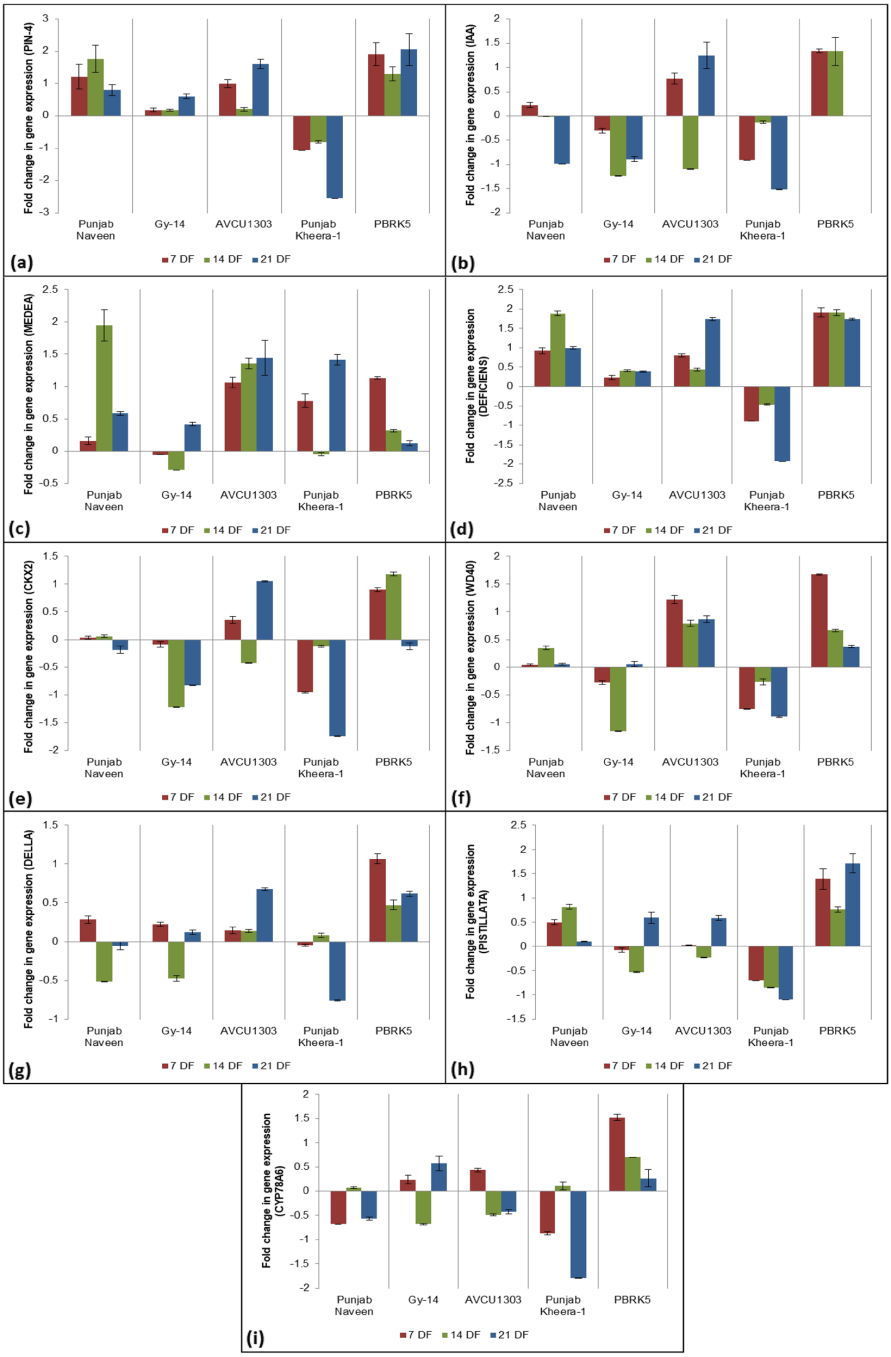
Fold changes in gene expression level of different parthenocarpy related genes at different days after flowering in cucumber (**a**) *CsPIN-4* (**b**) *CsIAA* (**c**) *CsMEDEA* (**d**) *CsDEFICIENS* (**e**) *CsCKX2* (**f**) *CsWD40* (**g**) *CsDELLA* (**h**) *CsPISTILLATA* (**i**) *CsCYP78A6*.

We examined the expression of nine PRGs (*CsPIN-4, CsIAA, CsMEDEA, CsDEFICIENS, CsCKX2, CsWD40, CsDELLA, CsPISTILLATA,* and *CsCYP78A6*) in five genotypes of cucumber including Gy-14 (gynoecious and non-parthenocarpic), AVCU1303 (sub-gynoecious and non-parthenocarpic), Punjab Naveen (monoecious and non-parthenocarpic), PBRK5 (monoecious and weak parthenocarpic) and Punjab Kheera-1 (gynoecious and parthenocarpic) using qRT-PCR (Fig. 10). Leaf samples were collected from each variety at 7, 14 and 21 days after flowering (DF). The nine PRGs chosen for validation were selected based on their function. All the genes selected were components of different pathways (phytohormone metabolism and signalling, reproductive development and regulation of biological processes) and had fallen under different GO terms (Table S2). The leaf samples were chosen for the study as a study conducted by Wang et al.^40^ revealed that the tissue specific expression of *GA20ox2* was the maximum in leaf sample of pear. *CsPIN-4* and *CsDEFECIENS* were down-regulated in parthenocarpic cucumber ‘Punjab Kheera-1’ with high fold changes (~ 2) (Fig. 10a and d). The genes *CsIAA, CsCKX2*, *CsWD40*, *CsDELLA*, *CsPISTILLATA* and *CsCYP78A6* were down-regulated in both parthenocarpic and non-parthenocarpic genotypes; however, the decrease in expression level was less in parthenocarpic as compared to non-parthenocarpic ones. The gene *CsMEDEA* was positively regulated in all genotypes except Gy14 (non-parthenocarpic) and Punjab Kheera-1(parthenocarpic) with slightly negative gene expression.

The fold change was the highest at 21 DF for all the genes except *CsPISTILLATA* in case of non-parthenocarpic genotype. The expression level was high at 7 DF, which decreased at 14 DF and again increased up to 21 DF. The inconsistency in the patterns followed by the expression level of the PRGs was consistent with the results of Li et al.^23^, Su et al.^24^ and Wu et al.^8^. In case of *CsPISTILLATA* gene, the expression level increased gradually from 7 to 21 DF. The genotype PBRK5 (parthenocarpic and monoecious) had all the genes positively expressed except *CsCKX2* with slight negative fold change (− 0.12) at 21 DF. Thus, the genes *CsPIN-4, CsWD-40* and *CsPISTILLATA* showed greater degree of fold change i.e. enhanced expression in parthenocarpic genotypes as compared to non-parthenocarpic genotypes. The intron–exon organization of the three genes showed high degree of variation in their genetic structure (Fig. 2). The genes *CsPIN-4, CsWD-40* and *CsPISTILLATA* were 2 kbp, 16 kbp and 3 kbp in length with 10, 18 and 6 exons respectively (Fig. 2). Moreover, the genes *CsPIN-4* and *CsPISTILLATA* were located under the homology group I and the gene *CsWD-40* was located in homology group IV tree (Fig. 5a). The genes *CsWD40* and *CsPIN-4* also showed parental polymorphism (Fig. 9) making them putative candidate genes for parthencarpy in cucumber.

## Discussion

Parthenocarpy comprises an important horticultural trait in many commercially grown fruit and vegetable crops. Due to its complex mechanism regulated by various genetic and environmental factors, the process of parthenocarpy is not completely understood in cucumber. Recent studies pertaining to parthenocarpy in cucumber included reports of major QTLs linked to parthenocarpy^24–26^ and transcriptome analysis of phytohormone biosynthesis and signal transduction genes^8^. The current study focused on the identification of genes regulating parthenocarpy via various pathways previously identified in other crop plants. A total of 35 PRG homologs were identified in cucumber which were distributed along all the seven chromosomes. Of all the genes, *CsPAT* and *CsMET1* were mapped in the regions of two cucumber parthenocarpy QTL, *parth4.1* and *parth5.1*, respectively^26^. This point lays down the foundation that there are many other genes which might plays significant role in regulation of parthenocarpy.

The comprehensive phylogenetic analysis was performed using Mega X software to understand the evolutionary significance of PRGs which clustered the genes into five homology groups on the basis of sequence similarity (Fig. 5a). The phylogenetic analysis showed that genes *DEFICIENS, PISTILLATA, SEP1, AGL6, MADS, AGAMOUS, FIE, GH3, PIN4, and MET1* were clustered in homology group I except *AtPISTILLATA, AtAGL6* and *SlAGL6*. The *AtPISTILLATA* was present in homology group IV along with *IAA* and *CYP78A6* genes and *AtAGL6* and *SlAGL6* were clustered in homology group V. The genes belonging to cucumber present in group I showed homology with the same genes of other plants, except *CsAGL6* which showed homology with *MADS* of *melon*, *citrus* and *tomato*. In previous reports, the *MADS* showed putative function in relation to flowering^41^. The MADS genes are considered homeotic genes and primarily their function was involved in determination of identification of flower concentric whorls^50^. Similarly *CsMEDEA* closely shared common clade with *CsLOG* gene whose function had been identified in expression of cytokinin biosynthesis genes related to anthesis^51^. It could be concluded that *CsAGL6* might have direct role in fertilization that related with seed development. The phylogenetic analysis showed that homology groups I and V were mainly related to fertilization independent seed formation and embryogenesis (Fig. 5a). However groups II and IV were related with genes activated with some other modification such as methytransferases and group III genes were related with auxin regulation. Previously, Wang et al.^34^ performed the phylogenetic analysis of *GA20ox2* gene in pear with that of *Arabidopsis*, apple, tomato, citrus, rice and grape depicting the *GA20ox2* to be closely linked with fertilisation. Similarly in current study revealed that *GA20ox2* gene in cucumber was closely linked to *melon* placed under group V (*CsFIS2, CsGID1, CsGA20OX, CsGA2ox1*) which implies this group might directly play role in fertilization and help to achieve the parthenocarpy in cucumber.

Intron–exon structure helps in identifying evolutionary changes. The exon–intron pattern of the DNA sequence was explored and plotted with the phylogenetic tree to provide some insight into the evolutionary gene structure. Further to understand the genic level structure, the genetic organization of the candidate group (homology group I and IV) was determined by analysis of intron and exon structure. The current study indicated that *CsSEP1* gene in cucumber (group I) comprised of seven exons (Fig. 2, and 5a). Yu et al.^52^ analysed the gene structure by studying the exon and intron pattern of *SEP1* and *SEP3* genes for agronomical traits, inferring that except for *SEP1/2*-like genes in *Brassicaceae*, all the genes had eight exons. *AGL6* subfamily (group I; Fig. 5a) consisted of two exons in cucumber (Fig. 2). The number of introns in coding sequences of *ARF* genes of *Vitis vinifera* and other family members range from one to three. In cucumber, *CsARF7* and *CsARF19* genes were predicted to have 12 introns. The result also revealed some variation in exons and introns number and length in different branches representing PRGs of homology groups I to V. In the homology group I, the number of exons and introns of cucumber PRGs ranged from 0 to 13 and 1 to 12 respectively (Fig. 2). They were also connected with the same common direct ancestor from melon and *Arabidopsis* (model plant) (Fig. 5a). The number of exons and introns gradually increased with increase in nodes and in homology group I, *CsFIE* had 13 exons and 12 introns. The overall analysis of phylogenetic tree and intron exon pattern revealed that group IV and V are highly impactful for the further study. The intron–exon structure pattern showed modification in the genes during evolution and the shuffling of intron in genes supported the neo-functionalization of genes across various taxa^52^.

The further evolutionary pattern and footprints were also checked by identifying conserved motifs. The conserved motifs were identified in cucumber PRGs (Fig. 4) which represented conserved sequences of amino acids across different genes whose function was assigned via GOMO analysis (Table 2). The result showed that the cucumber PRGs containing all the conserved protein motifs (motif 1–8) were present in homology groups I and IV (Table 2; Fig. 5a). According to phylogenetic analysis the groups I and IV, the genes *CsSEP1*, *CsPIN-4*, *CsIAA9* and *CsARF8* contained all eight types of motif pattern. However, the group III consisted of a few motifs with *CsYUCCA* having a single motif showing the loss of the protein motifs during evolution. The functional analysis of the motifs assigned their role in general plant metabolism except motif 4 (HARRAAAARAAAAGAAAAGRAAARRARA) which was involved in cytokinin mediated signalling pathway (GO:0,009,736). The motif was discovered in all genes except *CsIAA, CsAGL6, CsCYP78A6, CsYUCCA, CsMEDEA, CsDEFICIENS, CsIAA, CsARF7* and *CsLOG* (Fig. 4)*.* Thus, most of the genes were involved in phytohormone signalling pathways. The previous studies by Li et al.^53^, Fu et al.^54^, Su et al.^8^ and Sun et al.^55^ identified various conserved motifs in genes controlling parthenocarpy in plants such as *Arabidopsis*, grapevine and cucumber.

The transcriptional regulation can be better understood by the *Cis*-regulatory elements which are essential transcriptional regulatory units present in the promoter region of the sequence (Fig. 3). In the present study, 8 CREs were identified including CAAT box, G-box, BOX-4, GARE box, ABRE box, CArG box, IBOX and BOX-II. The CAAT box was the most abundant sequence that was present in all genes with the basic function of CAAT box being in endosperm or seed development^56^. The presence of CAAT box in all the homologs confirms that the motifs are conserved in plants. The G-box is one of the best characterized CREs in plants^57–59^. It plays an important role in fruit specific expression and has been identified in diverse set of unrelated genes, such as those regulated by visible and ultraviolet light^60^, ABA^61^, methyl-jasmonate and anaerobiosis and has a role in ethylene induction as well as in seed-specific expression. It is also known as ABRE (abscisic acid -responsive element)^62^. Studies have indicated that the G-box elements cannot act alone and require additional CREs for their function^63,64^. This statement supports the fact that the promoter region of cucumber PRGs contains a number of CREs required for the high and specific expression of the gene in fruit tissues^65^. Our study found that the G-box was located in *CsDELLA, CsFIE, CsMET1, CsARF19, CsPYR1, CsCKX2, CsAGL6, CsTPL* and *CsGAST1*. Their presence indicates the environmental factors such as light may play a role during parthenocarpic fruit formation. ABRE (CCACGTGG) motifs had been reported to be involved in abscisic acid regulation and are regulated by calcium^66,67^. ABRE related elements had also been detected in *A. thaliana* pathogenesis related sequences^68^. GARE motifs are gibberellin-responsive elements present^68^. The presence of ABRE and GARE motifs in the PRGs indicated that role of plant hormone signals’ crosstalk in the regulation of parthenocarpy. CArG constituted potential MADS domain protein binding sites regulating gynoecium development^69^. In vitro and in vivo assays had shown that MADS proteins bind as dimers to CArG boxes, with the consensus sequence CCA[A/T]6GG (SRF-type) or C[A/T]8G (MEF2-type)^70^. Certain MADS proteins such as AGAMOUS-LIKE-15 (AGL15) preferred longer MEF2- type binding site^71^. Besides CAAT box, the presence of such CREs which are regulated by light and hormonal interactions indicated that plant hormones and environmental factors interact with each other during fruit ripening process^72^. The findings suggest a complex network of regulation of parthenocarpy in cucumber.

Furthermore, the GO analysis was performed to categorize genes according to their origin/function. Biological process defines a gene based on its biological objective to which the gene or its product contributes; molecular function is defined as the biochemical activity of a gene product and cellular component refers to the cellular location where a gene product is active^73^. The genes *CsGA20OX* and *CsGA20OX2* were involved in gibberellin-20-oxidase activity (GO:0,045,544) (Table S3). Besides these two genes*, CsGA2ox1* was involved in gibberellin metabolic process (GO:0,009,685). The genes *CsCKX1* and *CsCKX2* were involved in cytokinin dehydrogenase activity (GO:0,019,139). The gibberellic acid (GA) synthesis or signaling genes have important roles in development of parthenocarpic fruit. In *Arabidopsis*, the overexpression of *GA2ox* induces seed abortion^74^. The gene is known to encode an enzyme which inactivates GA^75^. It has been previously reported that a deficiency of GAs leads to reduced seed growth due to poor utilization of assimilates^76^. The *CKX* genes involved in degradation of cytokinin were expressed less in highly parthenocarpic cucumber as compared to weak parthenocarpic cucumber which indicated that downregulation of *CKX* induced parthenocarpy^8^. Backiyarani et al.^77^ carried the GO analysis in *Musa* for parthenocarpy related genes. They showed that the majority of the genes were involved in regulation of cellular macromolecule biosynthesis process and transcriptional regulatory activity. In case of PRGs in zucchini (*Cucurbita pepo* L.), metabolic process and cellular component were the most represented groups^78^. Chen et al.^79^ performed GO analysis of the differentially expressed genes involved in parthenocarpy in case of eggplant. The majority of the genes belonged to membrane-bound organelle, DNA integration, RNA-directed DNA polymerase activity, nucleic and metabolic process, plasma membrane, and nucleic acid binding categories.

The expression profiles of various genes were studied. The genes *CsPIN-4, CsDEFICIENS* and *CsWD-40* were negatively expressed in Punjab Kheera-1 (Fig. 10a, d, f). Similar results were reported by Ong-Abdullah et al.^80^ who showed that loss-of-function mutation in *DEFICIENS* gene in *Elais guineensis* resulted in parthenocarpy. *DEFICIENS* had similar function to *PISTILLATA*; it is a B class *MADS*-box gene regulating petal/stamen identity in snapdragon^81^. Similarly, the loss of function of tomato *DEFICIENS* resulted in parthenocarpy, together with abnormal stamen differentiation^82^. The gene *PI* (*PISTILLATA*) is associated with parthenocarpic fruit development in apple (*Malus domestica*) but not in *Arabidopsis*^83^. The gene *CsDELLA* was negatively expressed at later stages in parthenocarpic genotypes (Punjab Kheera-1 and PBRK-5) (Fig. 10g). In tomato, the loss-of-function of *DELLA* gene (procera (pro) mutation) corresponding to a single non-synonymous substitution in the GRAS domain of the *SlDELLA* displayed enhanced gibberellic acid phenotypes including parthenocarpy^84^. The *PIN-FORMED (PIN)* protein family is responsible for auxin efflux transport and the *PIN* genes are involved in various developmental processes including embryogenesis, shoot and root morphogenesis, gravitropism, and phototropism^85^. In *Arabidopsis* stem cells, PIN regulates the expression of the WUSCHEL transcription factor, which indicates the importance of critical auxin gradient/transport to control vital root and shoot stem cell regulators^86^. Silencing of *SlPIN4* had been reported to cause precocious ovary development resulting in parthenocarpic fruit in tomato^87^. The gene *CsMEDEA* was positively regulated in all non-parthenocarpic genotypes and negatively regulated at intermediate stage in parthenocarpic genotypes. The gene encodes a polycomb group protein which is directly associated with promoter region of *PHE1* which is a MADS-box gene^21^. The *MEDEA* mutants had shown suppression in seed abortion indicating the expression of *MEDEA* as an essential regulator in seed development^21^. The WD40 repeat proteins play multiple roles in cellular processes, including cell cycle regulation, cell apoptosis, autophagy, gene transcription, signal transduction, histone modification, DNA damage repair, RNA modification, cytoskeletal assembly, and chromatin assembly^88^. The gene *CsWD40* has been described as an ortholog of *WD40* in *Arabidopsis* which plays important role in cytokinin response and has been described as a promising candidate gene related to parthenocarpy^89,90^. The reports were consistent with our study. The genes *CsWD-40* and *CsPIN-4* showing expression level also showed parental polymorphism using SSR markers. Thus, the genes *CsPIN-4* and *CsWD-40* could be considered as potential candidate genes to determine parthenocarpy.

### Conclusions

Although parthenocarpy is an important agronomic trait and has been used in production for a long time, the mechanisms of parthenocarpic fruit set seem complex. Though regulated by phytohormones, the mechanism is difficult to understand and the phenotypic demarcation is difficult as environmental factors play an important role in regulating fruit set. Thus, the study was carried out to identify the genes involved in parthenocarpy in cucumber. A total of 35 genes were identified via homology based approach in cucumber. Majority of the genes were involved in phytohormone synthesis, regulation and signalling. Phylogenetic analysis grouped the parthenocarpy related genes in different genera into five major homology groups clustering genes based on their functioning and phylogeny. The genes *CsDEFICIENS*, *CsPISTILLATA, CsWD40* and *CsPIN-4* were negatively expressed with high fold changes (~ 2) in parthenocarpic genotypes. Moreover, the genes *CsWD-40* and *CsPIN-4* also exhibited parental polymorphism. Thus these two genes could be used as candidate genes for determining parthenocarpy in cucumber.

## Materials and methods

### Identification and sequence retrieval of parthenocarpy related genes in cucumber

We reviewed the literature and identified PRGs from various fruit or vegetable crops that are either directly or indirectly involved in regulation of parthenocarpy. The crop plants included *Cucumis sativus* L. (cucumber)^8,24,26^, *Solanum lycopersicum* L. (tomato)^30,74,81–84^, *Pyrus communis* L*.* (pear)^44,85,86^, *Ficus carica* L. (fig)^33,87^; and across various other taxa^10,21,32,39,47,88^. Genomic DNA sequences of those PRGs in the cucumber genome were obtained through BLASTn in several databases Ensembl Plants, Cucurbits Genome Database and NCBI , which were further cross-verified by using BLASTp with default settings (expected threshold 0.05) and percentage identity more than 80% and e-value less than zero on query sequences using Ensembl Plant database (https://plants.ensembl.org/Multi/Tools/Blast). Only top hits were selected. The genes were plotted onto the seven chromosomes of cucumber in an orderly manner from the short-arm to the long-arm telomere using Phenogram Plot (http://visualization.ritchielab.org/phenograms/plot).

### Intron–exon gene structure of PRGs

Positions of exons and introns of these cucumber PRGs were determined based on genomic information. Full length genomic (gDNA) and coding sequences (CDS) of cucumber PRGs retrieved from EnsemblPlant (https://plants.ensembl.org/Multi/Tools/Blast) were further utilized for the determination of exon–intron organizations of these genes using Gene-Structure Display Server GSDS2.0 (https://gsds.cbi.pku.edu.cn)^91^.

### Cis-regulatory element analysis and identification of conserved motifs

*Cis*-regulating elements (CREs) of PRGs were analysed to explore the DNA binding domains in the promoter region. The genomic sequence of each gene (> 300 bp) upstream of the transcription start site was retrieved from NCBI database (https://www.ncbi.nlm.nih.gov/). The analysis of both sense and anti-sense strands of promoter sequences was carried out using Plant CARE (http://bioinformatics.psb.ugent.be/webtools/plantcare/html/)^92^ and PLACE (https://www.dna.affrc.go.jp/PLACE/?action=newplace)^93^. The conserved motifs were discovered using the MEME suite (https://meme-suite.org/meme/tools/meme)^94^ with parameters motif width ranging from 6 to 50 and number of sites in sequences for each motif ranging from 2 to 200. The maximum number of motifs to be found was set at 8. The function of each motif was further elucidated by submitting the motif sequence to GoMo (Gene ontology for motifs) version 5.5.0 (https://meme-suite.org/meme/tools/gomo)^95^ and significant threshold and number of scores shuffling rounds were set at 0.05 and 1000 respectively for annotation of the motifs.

### Phylogenetic analysis

The PRGs for phylogenetic analysis were considered from 5 different plants (*Arabidopsis,* melon, cucumber, tomato and citrus) each having 35 PRGs (total 175 PRGs). The nucleotide sequences of PRGs from *Arabidopsis*, melon, cucumber, tomato and citrus were aligned with gap opening and gap extension penalties of 10 and 0.1, respectively, using ClustalW. A Maximum-Likelihood method was used to develop a cladogram of all the sequences. The associated taxa clustered together in the bootstrap test of 1000 replicates. The phylogenetic tree was constructed using MEGA X software^96^ and visualized through iTOL Interactive Tree of Life (https://itol.embl.de/).

### Gene Ontology (GO) analysis and KEGG pathway annotation

The functional prediction of PRGs and the analysis of annotation data were done using BLAST2GO tool (https://www.blast2go/com/)^97^. The amino acid sequences of parthenocarpic genes were imported into BLAST2GO program to follow these three steps i.e., (i) BLASTP against protein database of NCBI (https://blast.ncbi.nlm.nih.gov/Blast.cgi) (ii) mapping and retrieval of gene ontology terms associated with BLAST search (iii) annotation of GO terms associated with each query to relate the sequences to known protein function. The Gene Annotation (GO) was categorized into three classes: cellular components, biological processes and molecular functions. The functional enrichment of the genes was performed using GProfiler (https://biit.cs.ut.ee/gprofiler/gost) using Bonferroni correction method with user threshold of 0.05 and numeric IDs treated as ENTREZGENE. Additionally, the KEGG mapping (https://www.kegg.jp/kegg/mapper/) was done to display enzymatic functions in the context of the metabolic pathways in which they participate^98^.

### Physical and chemical properties, homology modelling and protein–protein interaction network of PRG proteins

The physical and chemical properties of the proteins involved in parthenocarpy were examined using ProtParamExPasy server (https://web.expasy.org/protparam/)^99^. The properties included length, molecular weight, instability index, PI value, aliphatic index and Grand Average of Hydropathicity index (GRAVY). The sub-cellular location of the proteins was determined through ProtComp version 9.0 server (http://www.softberry.com/) and the Pfam domains were predicted via Pfam 35.0(http://pfam.xfam.org/) based on profile Hidden Markov Models^100^. The amino acid sequences of all the proteins were fed in Phyre2 (Protein Homology/analogY Recognition Engine; http://www.sbg.bio.ic.ac.uk/phyre2) for predicting the protein structure by homology modelling under ‘expert’ mode using HH-search alignment algorithm^101^. The search was performed in normal mode of Phyre2. The protein structure of all the proteins modelled at > 90% confidence. The conformational states of the proteins were predicted using SOPMA (https://npsa-prabi.ibcp.fr/cgi-bin/npsa_automat.pl?page=/NPSA/npsa_sopma.html) with output width 70, similarity threshold 8 and window width 17. The amino acid sequences were submitted to STRING v11.5 (https://string-db.org/), a pre-computed database for the exploration of protein–protein interaction (PPI) using STRING network type with medium confidence (0.400) and 5% false discover rate stringency. The PPI network was retrieved using k-means clustering with the maximum number of clusters set to five.

### SSR mining and evaluation

The SSRs were mined using MISA web tool (https://webblast.ipk-gatersleben.de/misa/)^102^. The coding sequences in FASTA format were uploaded in the MISA-web tool. A specific project name was specified and SSR search parameters were set as present by default. The file output parameter was generated as Misa. The primers for the SSRs were designed using PolyMorphPredict web-tool (http://webtom.cabgrid.res.in/polypred/)^103^. The list of the primers is provided in supplementary Table S1. The primes were procured from Integrated DNA Technologies Inc., USA. The amplification was performed using profile: initial denaturation at 95 °C for 5 min followed by 35 cycles of denaturation at 95 °C for 40 s, annealing for 40 s, extension at 72 °C for 40 s and final extention at 72 °C for 7 min and hold at 4 °C.

### qRT-PCR analysis of cucumber PRGs

Five cucumber lines with varying degrees of parthenocarpic fruit-set capacities were used to evaluate the association of the expression of PRGs. These genotypes included Gy-14 (gynoecious and non-parthenocarpic), AVCU1303 (sub-gynoecious and non-parthenocarpic), Punjab Naveen (monoecious and non-parthenocarpic), PBRK5 (monoecious and weak parthenocarpic) and Punjab Kheera-1 (gynoecious and parthenocarpic). The plants were grown under poly-house conditions (average temperature 30–35 °C). Three biological replicates for each genotype were taken for RNA isolation. The relative expression of selected PRGs was examined using quantitative real-time PCR (qRT-PCR). The leaf samples from test materials were collected at different time intervals: before flowering (control) and 7, 14 and 21 DF (Days after flowering). Total RNA was extracted using the Trizol™ reagent method and stored at − 80 °C. The cDNA was synthesized using the Thermo Scientific First Strand cDNA Synthesis Kit following manufacturer’s protocol. The quality and integrity of the total RNA and cDNA was checked via agarose gel electrophoresis and spectroscopic method using NanoDrop 2000D (NanoDrop Technologies, Wilmington, DE, USA).

The primers used for qRT-PCR were designed using Primer3 tool (https://bioinfo.ut.ee/primer3/) and validated for hairpin formation via OligoCalC (http://biotools.nubic.northwestern.edu/OligoCalc.html). Information of all primers used in this study is provided in supplemental Table S9. The cucumber 18S rRNA gene (GenBank ID: X51542.1), was used as an internal control^104^. qRT-PCR was performed with the KAPA SYBR FAST qPCR Master Mix kit (Kapa Biosystems). The relative gene expression was calculated using the 2^−ΔΔCT^ method following Livak and Schmittgen^105^. For each sample, there were three biological and three technical replicates.

## Supplementary Information

Supplementary Information 1.

Supplementary Information 2.

Supplementary Information 3.

## Data Availability

The datasets generated and/or analysed during the current study have been provided as either in the text or supplemental materials. The genomic DNA, cDNA sequences or deduced protein sequences are publicly available in the cucurbit genomics (https://www.cucurbitgenomics.org/) website.
