# Supplementary material for: Genome-wide identification and characterization of parthenocarpic fruit set-related gene homologs in cucumber (Cucumis sativus L.)
Source: Sci Rep. 2023 Feb 10;13:2403. doi: 10.1038/s41598-023-29660-3 (PMC9918540; doi:10.1038/s41598-023-29660-3)
Supplement: Supplementary file 1 — Supplementary Information 1. [file 41598_2023_29660_MOESM1_ESM.doc]

**Genome-wide identification and characterization of parthenocarpic fruit set-related gene homologs in cucumber (*Cucumis sativus* L.)**

Table S1: PRGs identified in various plants.

| **Organism** | **Gene** | **References** |
| --- | --- | --- |
| *Ficus carica* L. | *GA20ox* | [33, 34] |
|  | *GID* |  |
|  | *Aux/IAA* |  |
|  | *GH3* |  |
| *Pyrus communis* L. | *GA20ox* | [37] |
| *Pyrus bretshneideri* Rehd. | *CYP78A6* | [37] |
|  | *DEFICIENS* |  |
| *Cucumis sativus* L. | *CYP735A* | [8] |
|  | *LOG1* |  |
|  | *PYR1* |  |
|  | *IPT* |  |
|  | *CKX* |  |
|  | *WD40* | [24] |
|  | *EIN1* |  |
|  | *ARF19* |  |
| *Solanum lycopersicum* L. | *IAA9* | [42] |
|  | *ARF7* | [18] |
|  | *PIN4* | [84] |
|  | *AGAMOUS, AGL6* | [28, 29] |
|  | *TPL1* | [30] |
|  | *DELLA* | [27] |
|  | *SEP1* | [80] |
|  | *PAT* | [31, 34] |
|  | *RR16* | [50] |
|  | *MADS* | [41] |
|  | *ARF8* | [18] |
|  | *GAST* | [106] |
| *Eriobotrya japonica* | *YUCCA* | [29] |
| *Arabidopsis thaliana* | *MET1* | [39] |
|  | *MEDEA* | [21] |
|  | *FIE* | [22] |
|  | *FIS2* | [20] |
|  | *ARF8* | [18] |
| *Vitis vinifera* | *PISTILLATA* | [36] |

Table S2: GO analysis of the PRGs into categories: ‘Biological process’, ‘Molecular function’ and ‘Cellular component’

(Supplementary Table S2.xlsx)

Table S3: Functional enrichment analysis of cucumber PRGs

Supplementary Table S3.xlsx

Table S4: Proteins modelled using Phyre2

| **Query** | **Template proteins used for modelling** | **% Confidence** | **PDB header** | **PDB molecule** |
| --- | --- | --- | --- | --- |
| CsIPT | c6sa8A | 100 | De novo protein | Ring-like darpin-armadillo fusion h83-_d01 |
| CsDEFICIENS | c7nb0A | 99.8 | Plant protein | Developmental protein sepallata 3 |
| CsPISTILLATA | c4ox0D | 99.9 | Transcription | Developmental protein sepallata 3 |
| CsEIN1 | c6qrjA | 100 | Signalling protein | Hybrid kinase |
| CsIAA | c2q43A | 100 |  |  |
| CsFIS2 | c7ktpC | 100 | Gene regulation/ transferase | Polycomb protein suz12 |
| CsMEDEA | c4ri3A | 99.4 | Membrane protein | Photosystem II 2 kda protein |
| CsRR16 | c3gt7A | 99.9 | Hydrolase | Sensor protein |
| CsSEP1 | c4ox0D | 99.9 | Transcription | Developmental protein sepallata 3 |
| CsARF8, CsARF7 | c4lduA | 100 | DNA binding protein | Auxin response factor 5 |
| CsAGL6 | c7nb0A | 99.4 | Plant protein | Developmental protein sepallata 3 |
| CsGA20OX2 | c7ekdA | 100 | Biosynthetic protein | Gibberellin 3-beta-dioxygenase 2 |
| CsCKX2 | c4oalB | 100 | Oxidoreducatse | Cytokinin dehydrogenase 4 |
| CsWD40 | c6bm0B | 100 | Protein binding | Pre-mrna 3’ end processing protein wdr33 |
| CsTPL | c5c7eB | 100 | Transcription | Aspr 2 protein |
| CsGH3 | c5kodA | 100 | Ligase | Indole-3-acetic acid-amido synthetase gh3.5 |
| CsYUCCA | c7al4D | 100 | Flavoprotein | FAD dependent oxidoreducatse |
| CsMADS | d1mnmA | 99.9 | SRF like superfamily | - |
| CsGA2ox1 | c6ku3C | 100 | Biosynthetic protein | Gibberellin 2-beta-dioxygenase 3 |
| CsCYP735A1 | c5t6qA | 100 | Oxidoreducatse | Cytochrome p450 4b1 |
| CsPIN-4 | c7xxbA | 100 | Transport protein | Auxin efflux carrier component 3 |
| CsCYP78A6 | c5ylwA | 100 | Oxidoreducatse | Ferruginol synthase |
| CsPYR1 | c3k90C | 100 | Hormone receptor, hydrolase regulator | Putative uncharacterized molecule |
| CsAGAMOUS | c6ca8A | 90 | Isomerase | DNA topoisomerase 2 |
| CsPAT | c5wmiA | 100 | Transferase | Bifunctional aspartate aminotransferase |
| CsIAA | c2q43A | 100 | Hydrolase | IAA-amino acid hydrolase ilr1-like 2 |
| CsMET1 | c3av6A | 100 | Transferase | DNA (cytosine-5)-methyltransferase 1 |
| CsFIE | c2qxvA | 100 | Gene regulation | Embryonic ectoderm development |
| CsGAST1 | c5e5qB | 100 | Antimicrobial protein | Snakin-1 |
| CsGA20ox | c6lsvA | 100 | Plant protein | Probable 2-oxoglutarate-dependent dioxygenase |
| CsDELLA | c6kpdC | 100 | Transcription | Scarecrow-like protein 3 |
| CsLOG | c2q4oA | 100 | Structural genomics, unknown function | Uncharacterized protein |
| CsIAA9 | c2mukX | 100 | Transcription | Auxin-responsive protein IAA 17 |
| CsCKX1 | c4ml8Cc | 100 | Oxidoreductase | Cytokinin oxidase 2 |
| CsGID1 | c2zshA | 100 | Hormone receptor | Probable gibberellin receptor gid1l1 |

Table S5: Conformational states of PRG proteins

| **Gene name** | **α-helix (%)** | **Extended strand (%)** | **β-turn (%)** | **Random coil (%)** |
| --- | --- | --- | --- | --- |
| *CsYUCCA* | 30.93 | 16.98 | 6.74 | 45.35 |
| *CsDELLA* | 46.59 | 10.41 | 4.95 | 38.05 |
| *CsMEDEA* | 21.98 | 16.48 | 6.59 | 54.95 |
| *CsPIN-4* | 36.34 | 22.09 | 4.04 | 37.53 |
| *CsFIS2* | 35.33 | 13.16 | 4.85 | 46.65 |
| *CsPISTILLATA* | 69.23 | 4.20 | 4.90 | 21.68 |
| *CsDEFICIENS* | 57.28 | 19.42 | 7.77 | 15.53 |
| *CsFIE* | 8.38 | 34.86 | 9.19 | 47.57 |
| *CsGA20OX* | 32.28 | 17.20 | 5.82 | 44.71 |
| *CsMET1* | 33.55 | 17.03 | 5.87 | 43.55 |
| *CsSEP1* | 59.24 | 2.72 | 0.00 | 38.04 |
| *CsARF7* | 27.53 | 13.49 | 9.12 | 49.86 |
| *CsARF8* | 28.10 | 14.43 | 2.68 | 54.79 |
| *CsLOG* | 47.25 | 13.76 | 7.80 | 31.19 |
| *CsIPT* | 41.89 | 10.19 | 5.41 | 42.52 |
| *CsEIN1* | 49.86 | 12.70 | 4.86 | 32.57 |
| *CsWD40* | 14.35 | 18.89 | 6.88 | 59.88 |
| *CsCYP735A1* | 58.71 | 9.07 | 5.01 | 27.21 |
| *CsRR16* | 29.61 | 11.16 | 4.72 | 54.41 |
| *CsPYR1* | 37.95 | 17.86 | 6.25 | 37.95 |
| *CsCKX1* | 37.64 | 15.87 | 5.54 | 40.96 |
| *CsCKX2* | 31.57 | 17.97 | 5.30 | 45.16 |
| *CsMADS* | 41.71 | 13.37 | 4.81 | 40.11 |
| *CsGA20OX2* | 36.19 | 17.69 | 5.09 | 41.02 |
| *CsGA2OX1* | 28.87 | 19.35 | 4.76 | 47.02 |
| *CsIAA* | 40.14 | 16.78 | 5.67 | 37.41 |
| *CsIAA9* | 19.74 | 18.42 | 2.89 | 58.95 |
| *CsAGAMOUS* | 33.75 | 12.30 | 2.52 | 51.42 |
| *CsAGL6* | 59.87 | 10.53 | 6.58 | 23.03 |
| *CsTPL* | 22.21 | 23.78 | 7.93 | 46.08 |
| *CsGID1* | 29.69 | 21.88 | 5.62 | 42.81 |
| *CsGAST1* | 22.33 | 7.77 | 3.88 | 66.02 |
| *CsPAT* | 42.11 | 16.21 | 6.32 | 35.37 |
| *CsCYP78A6* | 51.59 | 9.72 | 4.86 | 33.83 |
| *CsGH3* | 39.53 | 14.45 | 5.65 | 40.37 |

Table S6: Percentage composition of essential amino acids in PRG proteins.

| **Gene name** | **% Histidine** | **% Isoleucine** | **% Leucine** | **% Lysine** | **% Methionine** | **% Phenylalanine** | **% Threonine** | **% Tryptophan** | **% Valine** |
| --- | --- | --- | --- | --- | --- | --- | --- | --- | --- |
| *CsYUCCA* | 2.8 | 5.6 | 8.4 | 7.4 | 2.1 | 4.9 | 3.3 | 1.9 | 9.1 |
| *CsDELLA* | 3.2 | 3.6 | 10.2 | 4.1 | 3.2 | 3.9 | 3.9 | 1.2 | 7.0 |
| *CsMEDEA* | 0.0 | 4.9 | 8.2 | 5.5 | 1.1 | 7.7 | 5.5 | 0.5 | 2.2 |
| *CsPIN-4* | 1.0 | 9.7 | 14.7 | 3.8 | 2.6 | 4.3 | 5.0 | 1.9 | 8.8 |
| *CsFIS2* | 3.9 | 3.0 | 8.8 | 6.0 | 2.8 | 5.5 | 3.7 | 1.4 | 6.9 |
| *CsPISTILLATA* | 2.8 | 3.5 | 8.4 | 7.0 | 8.4 | 2.8 | 2.8 | 0.7 | 4.2 |
| *CsDEFICIENS* | 2.4 | 7.3 | 9.2 | 11.2 | 3.4 | 3.4 | 7.3 | 1.5 | 5.8 |
| *CsFIE* | 1.9 | 7.8 | 5.7 | 5.9 | 1.6 | 5.4 | 4.9 | 3.0 | 9.5 |
| *CsGA20OX* | 4.2 | 2.6 | 10.1 | 7.4 | 1.6 | 7.1 | 4.5 | 1.6 | 6.3 |
| *CsMET1* | 1.5 | 5.6 | 7.9 | 8.0 | 2.6 | 3.9 | 4.5 | 1.4 | 6.0 |
| *CsSEP1* | 0.5 | 3.3 | 13.6 | 6.0 | 2.7 | 2.2 | 8.2 | 1.1 | 3.3 |
| *CsARF7* | 2.4 | 3.6 | 10.0 | 3.6 | 2.1 | 3.8 | 4.9 | 1.3 | 4.6 |
| *CsARF8* | 3.7 | 3.7 | 9.6 | 2.6 | 2.8 | 4.3 | 5.4 | 1.7 | 6.3 |
| *CsLOG* | 3.2 | 6.9 | 7.8 | 6.9 | 3.2 | 2.3 | 2.8 | 0.9 | 9.2 |
| *CsIPT* | 3.4 | 5.0 | 8.9 | 5.3 | 2.0 | 3.5 | 4.0 | 1.7 | 5.5 |
| *CsEIN1* | 3.0 | 5.9 | 13.1 | 4.9 | 3.1 | 4.1 | 6.5 | 0.9 | 9.6 |
| *CsWD40* | 4.5 | 2.5 | 5.9 | 4.2 | 4.7 | 3.8 | 5.1 | 2.5 | 4.7 |
| *CsCYP735A1* | 2.9 | 5.0 | 15.0 | 6.2 | 3.6 | 5.0 | 5.5 | 1.9 | 4.5 |
| *CsRR16* | 0.9 | 5.2 | 8.2 | 4.7 | 3.0 | 1.7 | 4.7 | 0.0 | 7.3 |
| *CsPYR1* | 3.6 | 4.0 | 9.4 | 3.1 | 1.3 | 3.1 | 6.2 | 0.9 | 8.0 |
| *CsCKX1* | 3.3 | 8.3 | 10.3 | 5.2 | 1.7 | 5.9 | 4.4 | 1.9 | 5.4 |
| *CsCKX2* | 3.2 | 6.2 | 10.4 | 4.6 | 1.4 | 4.4 | 5.1 | 2.1 | 8.1 |
| *CsMADS* | 1.6 | 5.3 | 10.2 | 8.0 | 2.7 | 7.5 | 5.3 | 1.1 | 5.9 |
| *CsGA20OX2* | 3.8 | 2.7 | 9.7 | 6.2 | 2.9 | 7.2 | 3.5 | 1.1 | 7.2 |
| *CsGA2OX1* | 2.7 | 6.8 | 11.3 | 7.4 | 1.8 | 6.0 | 3.9 | 1.2 | 5.1 |
| *CsIAA* | 3.6 | 4.5 | 9.1 | 3.9 | 2.5 | 4.5 | 5.0 | 1.4 | 9.1 |
| *CsIAA9* | 2.1 | 2.6 | 9.2 | 6.8 | 2.4 | 2.9 | 4.2 | 1.3 | 5.8 |
| *CsAGAMOUS* | 2.5 | 3.2 | 11.7 | 4.4 | 3.5 | 3.5 | 4.7 | 0.9 | 2.8 |
| *CsAGL6* | 0.7 | 3.9 | 13.2 | 11.8 | 4.6 | 2.6 | 0.7 | 0.7 | 5.9 |
| *CsTPL* | 2.3 | 4.3 | 8.7 | 5.6 | 2.1 | 4.7 | 5.4 | 1.8 | 7.0 |
| *CsGID1* | 4.4 | 4.7 | 10.9 | 4.4 | 2.2 | 6.2 | 1.2 | 1.9 | 6.9 |
| *CsGAST1* | 1.9 | 1.0 | 6.8 | 8.7 | 2.9 | 2.9 | 7.8 | 1.0 | 3.9 |
| *CsPAT* | 1.9 | 6.3 | 9.7 | 5.3 | 1.5 | 3.8 | 4.6 | 0.6 | 7.8 |
| *CsCYP78A6* | 2.2 | 5.8 | 12.1 | 3.4 | 3.0 | 4.7 | 5.2 | 2.4 | 7.3 |
| *CsGH3* | 1.3 | 6.8 | 10.0 | 6.5 | 1.8 | 4.2 | 6.6 | 0.8 | 6.5 |

Table S7: List of SSRs mined from cDNA, coding and genomic sequences of parthenocarpy related genes in cucumber

| **SSRs from cDNA sequences** | | | | | | |
| --- | --- | --- | --- | --- | --- | --- |
| **Gene** | **SSR nr.** | **SSR type** | **SSR** | **size** | **start** | **end** |
| *CsDELLA* | 1 | p4 | (AGAA)7 | 28 | 110 | 137 |
|  | 2 | c* | (T)14ctctttcctttttttggtggatatcccagggctcttttgtaattttcaatggtaaatttc(A)11(AAG)7* | 104 | 2462 | 2565 |
| *CsPIN-4* | 1 | p3 | (ATG)6 | 18 | 900 | 917 |
| *CsFIS2* | 1 | p1 | (T)10 | 10 | 927 | 936 |
|  | 2 | p1 | (T)11 | 11 | 1343 | 1353 |
| *CsARF8* | 1 | p3 | (CAG)5 | 15 | 1462 | 1476 |
| *CsEIN1* | 1 | p1 | (T)11 | 11 | 363 | 373 |
| *CsRR16* | 1 | p1 | (A)10 | 10 | 1225 | 1234 |
| *CsPYR1* | 1 | p3 | (TTC)5 | 15 | 792 | 806 |
| *CsGA2ox* | 1 | p2 | (AG)7 | 14 | 1077 | 1090 |
| *CsIAA* | 1 | p3 | (TTC)5 | 15 | 519 | 533 |
| *CsINO* | 1 | p3 | (TTC)5 | 15 | 519 | 533 |
| *CsIAA9* | 1 | p1 | (T)17 | 17 | 127 | 143 |
|  | 2 | p1 | (T)16 | 16 | 383 | 398 |
| *CsTPL* | 1 | p1 | (T)12 | 12 | 3343 | 3354 |
| *CsGAST1* | 1 | p1 | (A)11 | 11 | 338 | 348 |
| **SSRs from coding sequences** | | | | | | |
| **Gene** | **SSR nr.** | **SSR type** | **SSR** | **size** | **start** | **end** |
| *CsPIN-4* | 1 | p3 | (ATG)6 | 18 | 626 | 643 |
| *CsARF8* | 1 | p3 | (CAG)5 | 15 | 1462 | 1476 |
| *CsIAA* | 1 | p3 | (TTC)5 | 15 | 330 | 344 |
| **SSRs from genomic sequences** | | | | | | |
| **Gene** | **SSR nr.** | **SSR type** | **SSR** | **size** | **start** | **end** |
| *CsDELLA* | 1 | p4 | (AGAA)7 | 28 | 110 | 137 |
|  | 2 | c* | (T)14ctctttcctttttttggtggatatcccagggctcttttgtaattttcaatggtaaatttc(A)11(AAG)7* | 104 | 2462 | 2565 |
| *CsPIN-4* | 1 | p1 | (T)17 | 17 | 421 | 437 |
|  | 2 | p2 | (TA)8 | 16 | 665 | 680 |
|  | 3 | p3 | (ATG)6 | 18 | 1333 | 1350 |
| *CsFIS2* | 1 | p1 | (T)10 | 10 | 927 | 936 |
|  | 2 | p1 | (T)11 | 11 | 1343 | 1353 |
|  | 3 | p2 | (TG)6 | 12 | 3412 | 3423 |
|  | 4 | p1 | (T)12 | 12 | 6040 | 6051 |
| *CsPISTILLATA* | 1 | C | (TTC)5tttggatatgtgagaaatgaaaccctaaaagaaag(TA)8 | 66 | 283 | 348 |
|  | 2 | p1 | (T)10 | 10 | 581 | 590 |
|  | 3 | p1 | (T)11 | 11 | 722 | 732 |
|  | 4 | p1 | (T)10 | 10 | 1135 | 1144 |
|  | 5 | p2 | (AT)9 | 18 | 1656 | 1673 |
|  | 6 | p1 | (A)10 | 10 | 1851 | 1860 |
|  | 7 | p1 | (T)13 | 13 | 2029 | 2041 |
|  | 8 | p1 | (T)11 | 11 | 2757 | 2767 |
| *CsDEFICIENS* | 1 | C | (AT)6aataaatcaaatcaaccacaccctatccca(TTC)5 | 57 | 286 | 342 |
|  | 2 | p1 | (T)10 | 10 | 3150 | 3159 |
|  | 3 | p2 | (TA)6 | 12 | 3353 | 3364 |
| *CsFIE* | 1 | p1 | (A)11 | 11 | 1236 | 1246 |
| *CsMET1* | 1 | p2 | (TA)7 | 14 | 2998 | 3011 |
|  | 2 | p2 | (TA)6 | 12 | 6897 | 6908 |
| *CsSEP1* | 1 | p4 | (TTTC)5 | 20 | 411 | 430 |
|  | 2 | p2 | (TA)8 | 16 | 1087 | 1102 |
|  | 3 | p1 | (A)14 | 14 | 2566 | 2579 |
|  | 4 | p1 | (T)23 | 23 | 4232 | 4254 |
|  | 5 | p1 | (T)11 | 11 | 4773 | 4783 |
|  | 6 | p2 | (AT)9 | 18 | 6137 | 6154 |
| *CsARF8* | 1 | p1 | (T)11 | 11 | 399 | 409 |
|  | 2 | p1 | (T)10 | 10 | 10099 | 10108 |
|  | 3 | p1 | (T)12 | 12 | 12235 | 12246 |
|  | 4 | p1 | (T)10 | 10 | 13180 | 13189 |
|  | 5 | p2 | (CA)9 | 18 | 13666 | 13683 |
|  | 6 | p1 | (T)13 | 13 | 17942 | 17954 |
|  | 7 | p3 | (CAG)5 | 15 | 20634 | 20648 |
| *CsLOG* | 1 | p1 | (C)20 | 20 | 145 | 164 |
|  | 2 | p2 | (CA)7 | 14 | 876 | 889 |
|  | 3 | p1 | (T)10 | 10 | 2485 | 2494 |
| *CsIPT* | 1 | p1 | (T)10 | 10 | 3227 | 3236 |
|  | 2 | p1 | (T)10 | 10 | 6862 | 6871 |
|  | 3 | p2 | (AC)6 | 12 | 8194 | 8205 |
| *CsEIN1* | 1 | p1 | (T)11 | 11 | 363 | 373 |
|  | 2 | p1 | (T)11 | 11 | 2469 | 2479 |
| *CsWD40* | 1 | p2 | (TA)6 | 12 | 138 | 149 |
|  | 2 | p2 | (AT)9 | 18 | 938 | 955 |
|  | 3 | p1 | (A)11 | 11 | 2787 | 2797 |
|  | 4 | p1 | (A)12 | 12 | 3926 | 3937 |
|  | 5 | p1 | (A)17 | 17 | 4138 | 4154 |
|  | 6 | p1 | (T)10 | 10 | 7974 | 7983 |
|  | 7 | p1 | (A)16 | 16 | 12648 | 12663 |
| *CsCYP735A1* | 1 | p1 | (T)14 | 14 | 998 | 1011 |
|  | 2 | p1 | (T)11 | 11 | 2073 | 2083 |
|  | 3 | p1 | (T)10 | 10 | 3879 | 3888 |
|  | 4 | p1 | (T)18 | 18 | 4025 | 4042 |
| *CsRR16* | 1 | p1 | (T)14 | 14 | 560 | 573 |
|  | 2 | p1 | (A)10 | 10 | 1502 | 1511 |
| *CsIAA* | 1 | p5 | (GAAAA)36 | 180 | 2723 | 2902 |
|  | 2 | p1 | (T)12 | 12 | 5925 | 5936 |
|  | 3 | p3 | (ATT)5 | 15 | 7780 | 7794 |
|  | 4 | p1 | (A)10 | 10 | 9890 | 9899 |
|  | 5 | p2 | (CA)8 | 16 | 11172 | 11187 |
|  | 6 | p1 | (T)10 | 10 | 14127 | 14136 |
| *CsPYR1* | 1 | p3 | (TTC)5 | 15 | 792 | 806 |
| *CsCKX2* | 1 | p2 | (AC)9 | 18 | 1997 | 2014 |
|  | 2 | p2 | (TA)60 | 120 | 2302 | 2421 |
|  | 3 | c | (T)16atatttaacattaaacaataaactaccaaattat(TC)9 | 68 | 3033 | 3100 |
| *CsGA2ox1* | 1 | p3 | (CTT)8 | 24 | 946 | 969 |
|  | 2 | p2 | (AG)7 | 14 | 1481 | 1494 |
| *CsIAA* | 1 | c* | (TTC)5cacacctgtttttggtctcagagcagacatggatgcccttcctcttcaggtactaatatctctctctttatatatacata(TC)7(TCT)7* | 128 | 519 | 646 |
| *CsIAA9* | 1 | p1 | (T)17 | 17 | 127 | 143 |
|  | 2 | p1 | (T)16 | 16 | 383 | 398 |
| *CsAGAMOUS* | 1 | p1 | (T)11 | 11 | 1270 | 1280 |
|  | 2 | p1 | (A)10 | 10 | 1927 | 1936 |
| *CsTPL* | 1 | p1 | (T)10 | 10 | 118 | 127 |
|  | 2 | p1 | (T)12 | 12 | 6655 | 6666 |
| *CsGID1* | 1 | p1 | (T)16 | 16 | 2029 | 2044 |
|  | 2 | p1 | (T)11 | 11 | 2267 | 2277 |
|  | 3 | p1 | (T)14 | 14 | 2464 | 2477 |
|  | 4 | p3 | (TTC)5 | 15 | 4235 | 4249 |
| *CsGAST1* | 1 | p2 | (AT)7 | 14 | 131 | 144 |
|  | 2 | p1 | (A)11 | 11 | 800 | 810 |
| *CsCYP78A6* | 1 | p1 | (A)10 | 10 | 1011 | 1020 |
| *CsGH3* | 1 | p1 | (T)13 | 13 | 435 | 447 |
|  | 2 | p1 | (T)10 | 10 | 2463 | 2472 |

Table S8: List of primer pairs 5’-3’ for SSR markers

| **Gene** | **Primer** | **SEQUENCE** |
| --- | --- | --- |
| *CsDELLA* | Froward primer | GCCGGGAGAAGGTGATTCAA |
|  | Reverse primer | CAGCGGCGGAAAAGGAAAAA |
| *CsDELLA* | Froward primer | CGCTTGGAAAATCGGCAACA |
|  | Reverse primer | AAGGTGAGGGTGAAAGGCAC |
| *CsPIN-4* | Froward primer | TTGTGGGTGTGGGTTTGTGA |
|  | Reverse primer | TGAGGAGGAGGTTTGGTCCA |
| *CsPIN-4* | Froward primer | GTAGGCTTCAGCTGCTGTCA |
|  | Reverse primer | GGCAGCTGAGTCATCGATGA |
| *CsFIS2* | Froward primer | TATGAGTGGTGCATGTGCGT |
|  | Reverse primer | TGACCGGGAAATACATGAACGT |
| *CsPISTILLATA* | Froward primer | AGGAGATGGTTGCAATGGGA |
|  | Reverse primer | GGCTGAATAGGCTGAACTCTGA |
| *CsPISTILLATA* | Froward primer | TCGTGAGAAGCAGGTTTTTCCT |
|  | Reverse primer | CCGACTGCAACCACAAAACC |
| *CsDEFICIENS* | Froward primer | ATGGTGCGGTCCAGAGAATG |
|  | Reverse primer | ACCTTGCGTTCACGGATGAT |
| *CsDEFICIENS* | Froward primer | GGGCTGGTAGACAATGGAGG |
|  | Reverse primer | ATGGTTGTTGTGGGTGTGGT |
| *CsMET1* | Froward primer | TGCGCCGAATTTTGCTTTCA |
|  | Reverse primer | CCGACCTAACCCAATGCAGT |
| *CsMET1* | Froward primer | TGTGAAGGTGTGGTTGCCTT |
|  | Reverse primer | AGACCCCCACAACACGTTTT |
| *CsSEP1* | Froward primer | CCTCCCTGGTTGGATGCTTT |
|  | Reverse primer | AGGCCAAAGAGTCAAATTATTGGC |
| *CsSEP1* | Froward primer | AAGCCCTTCCTTGGAAATCA |
|  | Reverse primer | GTTTATGCACCCGCCTTTGG |
| *CsSEP1* | Froward primer | ACATCTGTCTGCCTTATGGGAC |
|  | Reverse primer | AGGACTGCGAGACAATTCAAGT |
| *CsARF8* | Froward primer | CCACACACAACCACACACAC |
|  | Reverse primer | CCTCATGCCAGGCAGAAGAA |
| *CsARF8* | Froward primer | CCATGTTGGCTGCTGGAATG |
|  | Reverse primer | TTATGGAAGCCTGCCTGTGG |
| *CsLOG* | Froward primer | GCTGAGATGGCCAGGAGATC |
|  | Reverse primer | CTCTAAAGCTTGGGGCCCAA |
| *CsIPT* | Froward primer | ACCCAACACACACACACTGT |
|  | Reverse primer | TGCATGTCACGAGGCATAGG |
| *CsWD40* | Froward primer | GTCCTGCAGCTTTTGGTTGG |
|  | Reverse primer | GGTTCTTGAACGTGTGCACC |
| *CsWD40* | Froward primer | TCCCTCTTCTTTTGTGCCCG |
|  | Reverse primer | ATTTGCAAGGACCGTGGCTA |
| *CsARF19* | Froward primer | TACAGCTACGAGGGCTTTGC |
|  | Reverse primer | GCCAATGGCCTGTTAGCATG |
| *CsARF19* | Froward primer | AGCAAAGGTGAGGCAATGGA |
|  | Reverse primer | TCCCAACACCGTTGTTCACA |
| *CsARF19* | Froward primer | ATGAATCCGCAGCTGGTGAA |
|  | Reverse primer | CAGGGGAAGAAGCACTCCTG |
| *CsPYR1* | Froward primer | GCTCGTTTGTTCGCCGATAC |
|  | Reverse primer | TTCCGTTTATGCCCTCCACC |
| *CsCKX2* | Froward primer | TTAAGGGGAAGTGGGCCAAC |
|  | Reverse primer | TGGGTGGGTGTATGTGTGTG |
| *CsCKX2* | Froward primer | AGTGGAGGCGTTGATGAAGG |
|  | Reverse primer | TAACTCGGCCTTGTGAACCC |
| *CsGA2ox2* | Froward primer | AGGAGATGGCTTGCCAAGTG |
|  | Reverse primer | CAAAGCTTCAAGTTCCGGGC |
| *CsGA2ox2* | Froward primer | GAATCGCTTTCTCCCTCCCC |
|  | Reverse primer | TTCATTCAATGGTGGCCCAC |
| *CsIAA* | Froward primer | GGCGAGGGAAAGGGAATTCA |
|  | Reverse primer | AAGGGCATCCATGTCTGCTC |
| *CsGID1* | Froward primer | CACACACAACCCCTTCCCAA |
|  | Reverse primer | TCAGAAGAGGCGTTCACGAA |
| *CsGAST1* | Froward primer | AAGGCAGCCTTCAACCTCAA |
|  | Reverse primer | CTGTGCATCTAGCCCCACAT |

Table S9: List of primer pairs 5’-3’ used for qRT-PCR

| **Gene** | **Primer** | **Sequence** |
| --- | --- | --- |
| *CsIAA* | Froward primer | CGTACAGCCCTCCTTACCAA |
|  | Reverse primer | AAATGCAACTGTCACCACCC |
| *CsDELLA* | Froward primer | GGGAGCATCACCATCTTCAT |
|  | Reverse primer | CCCAAAACAGCAAGAAGCTC |
| *CsMEDEA* | Froward primer | CATTCCCCTTCCCCAGTATC |
|  | Reverse primer | GGTCTGAGAGCTTCGAATGG |
| *CsPIN-4* | Froward primer | CTTCCATTCCCGTTCTGAAA |
|  | Reverse primer | AGCAAGATTGGAGGAGACGA |
| *CsPISTILLATA* | Froward primer | GGGATGCTAAGCATGAGAA |
|  | Reverse primer | TTTTCATGAATTCCGACTGC |
| *CsDEFICIENS* | Froward primer | GTATACCACCCCTGCCACTT |
|  | Reverse primer | GCCTAATCTGCCTTCGGAGA |
| *CsWD40* | Froward primer | CCTTTGGAATGGACAGTCGT |
|  | Reverse primer | CAGAAGCTCAAGTCCCGAAC |
| *CsCKX2* | Froward primer | AGGCGTTGATGAAGGAATTG |
|  | Reverse primer | CTCTTCGTCCCAAAGTCGAG |
| *CsCYP78A6* | Froward primer | ATTTTCTATTGGGCCCATCC |
|  | Reverse primer | GAGTGTTGGCAAGGGAAAAA |


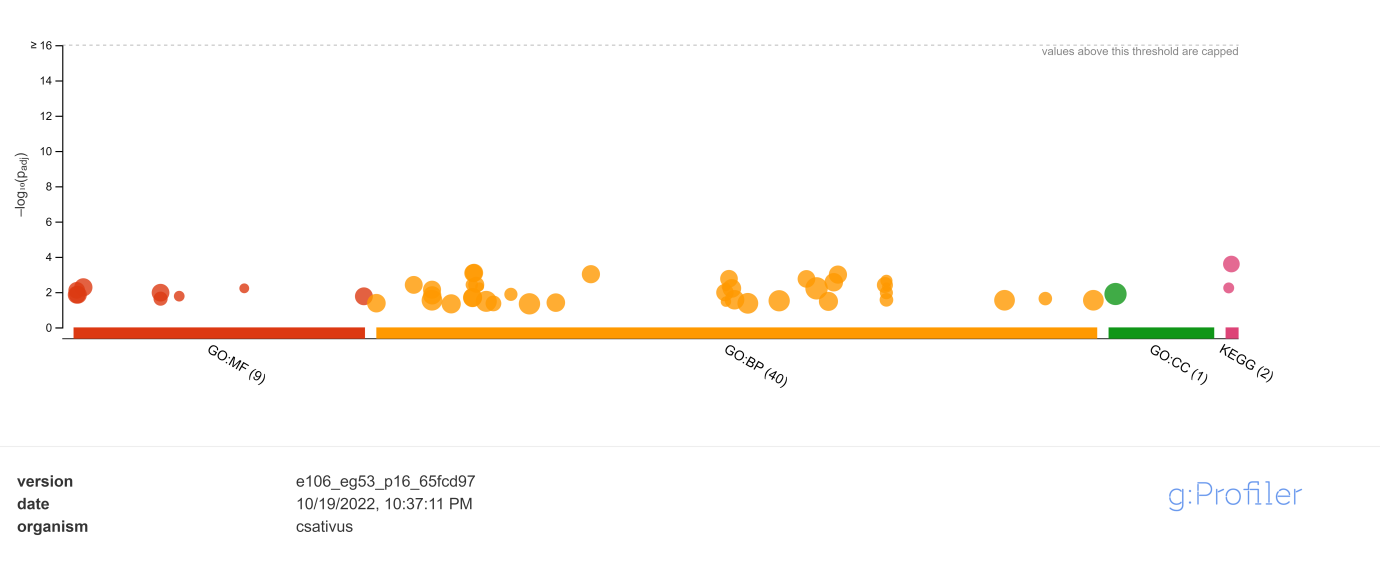


Fig. S1: Functional enrichment of cucumber PRGs (MF: Molecular function; BP: Biological Process; CC: Cellular component)

*
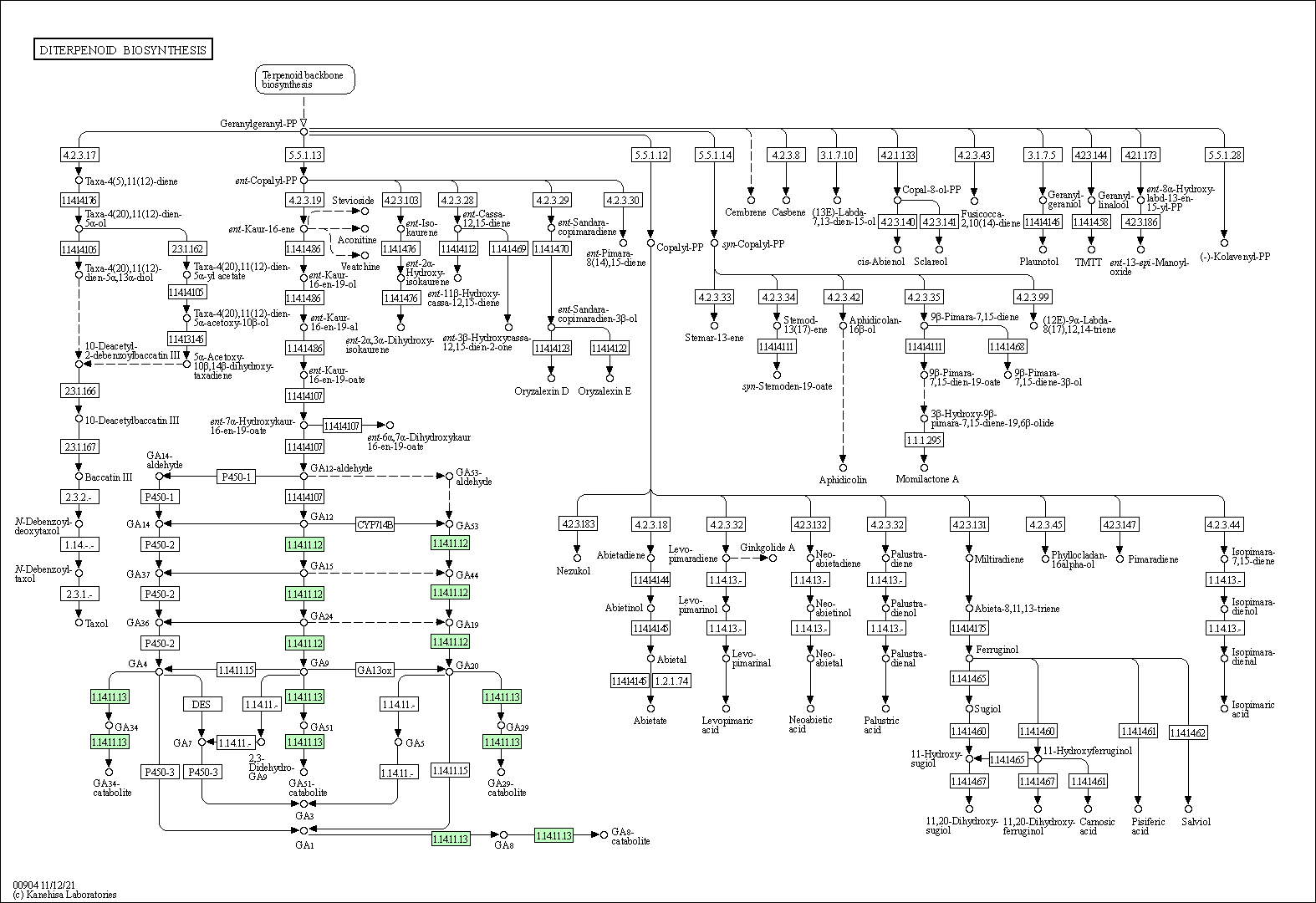
*

FigS2(a): Proteins involved in diterpenoid biosynthesis (KEGG pathway map00904 [98])

*
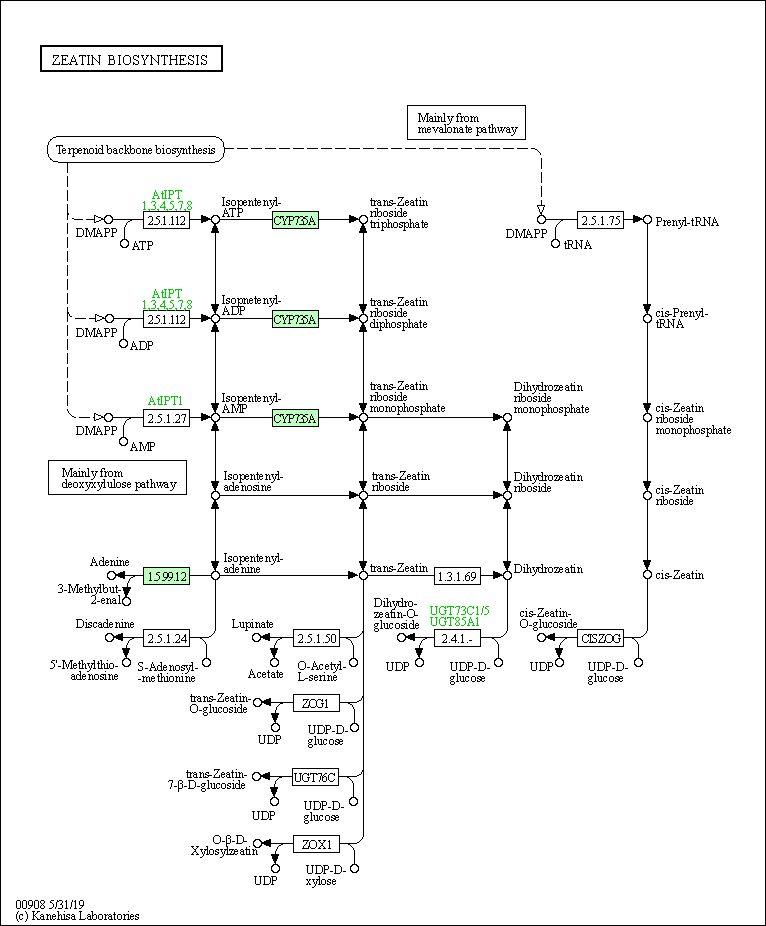
*

FigS2(b): Proteins involved in zeatin biosynthesis (KEGG pathway map00908 [98])

*
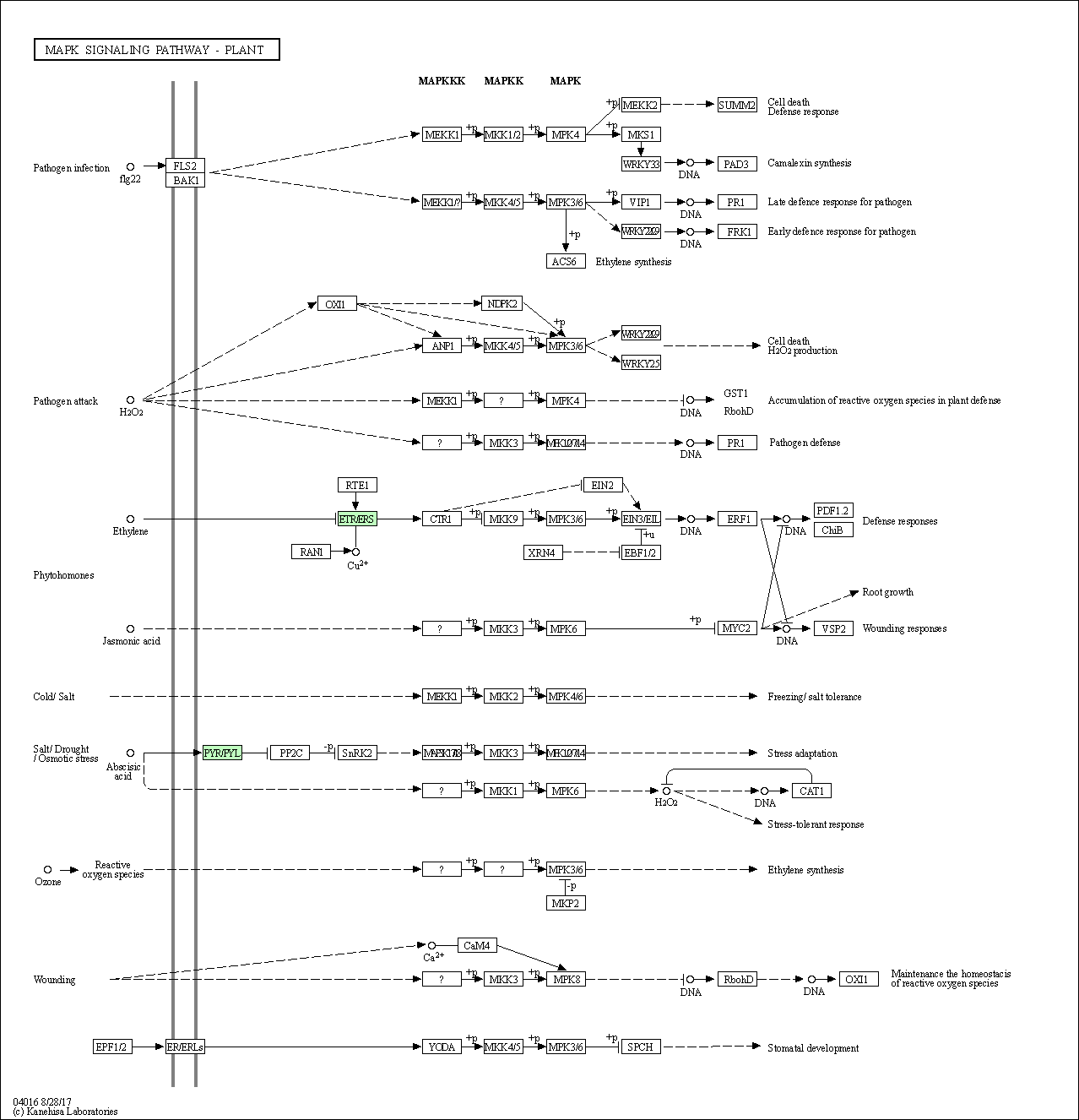
*

FigS2(c): Proteins involved in MAPK signalling pathway (KEGG pathway map04016 [98])

*
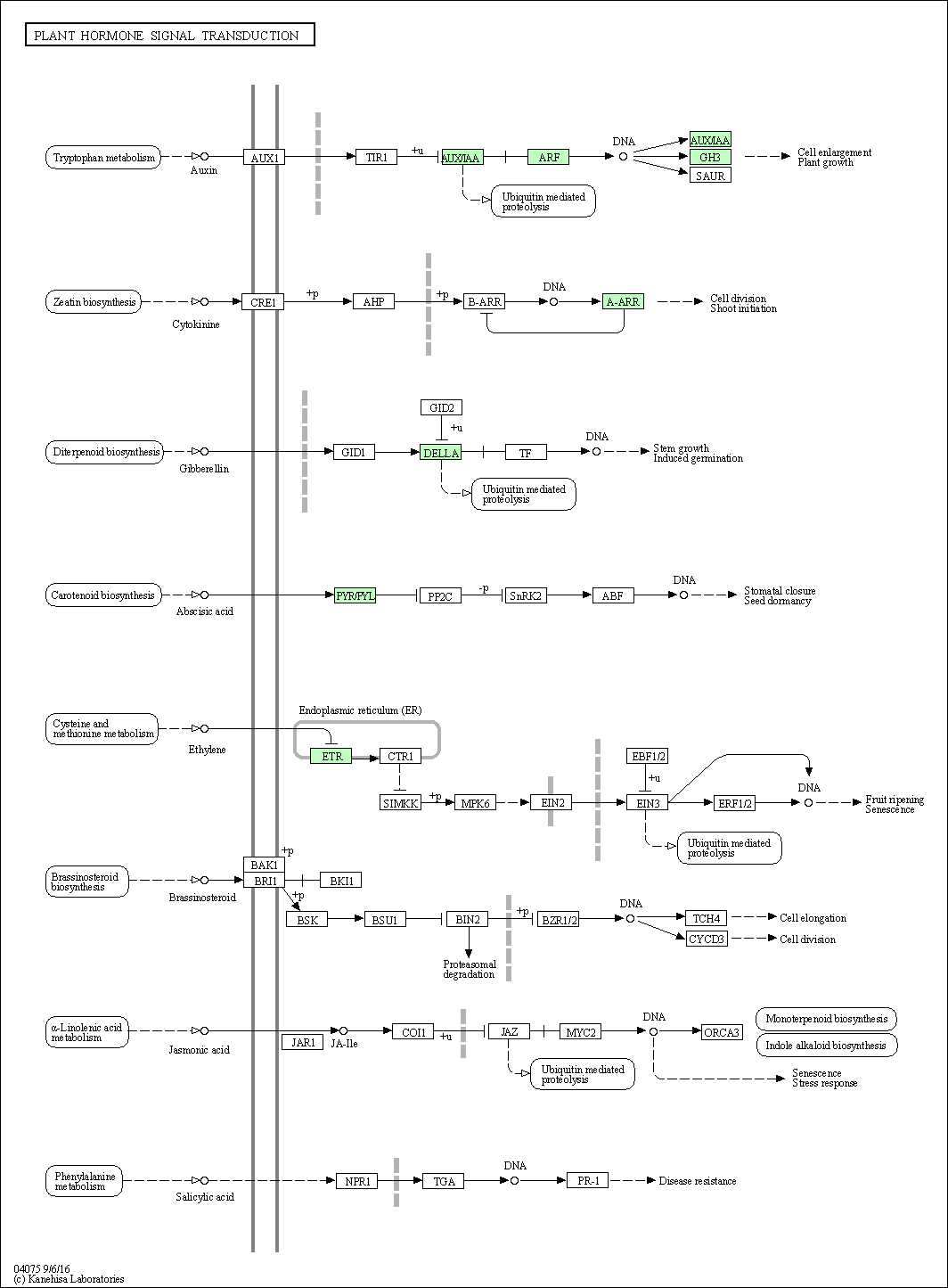
*

FigS2(d): Proteins involved in plant hormone signal transduction (KEGG pathway map04075 [98])
